# Supplementary material for: Europeans support large carnivore recovery while opposing both further population growth and hunting
Source: Nat Ecol Evol. 2025 Nov 25;10(1):28–33. doi: 10.1038/s41559-025-02914-1 (PMC12789005; doi:10.1038/s41559-025-02914-1)
Supplement: Supplementary file 1 — Supplementary Methods, Results, Survey Translations and References. [file 41559_2025_2914_MOESM1_ESM.pdf]

nature ecology & evolution

Supplementary information https://doi.org/10.1038/s41559-025-02914-1

Europeans support large carnivore recovery while opposing both further population growth and hunting

In the format provided by the authors and unedited

Supplementary Methods

Panelist recruitment

Qualtrics uses several sources for its panel aggregation (e.g., targeted emails, website intercepts, social media), and places caps on how many can come from a specific recruitment source to prevent a bias from entering data. Each of the individual panelists are vetted further by a proprietary technology which performs a digital fingerprint check to ensure people are who they say they are. Panelists are then invited to participate in surveys via email informing them that they will be compensated (the type and extent of compensation may vary). Qualtrics applies additional quality control measures to exclude responses from participants who completed the survey too quickly or exhibited patterned or non-serious answering behavior. The common limitations of such non-probability samples (i.e. not all individual in the targeted population have a known and non-zero probability to be sampled) apply to our study [1]. It is possible, for example, that individuals with a higher level of education may be overrepresented in our panels versus the population, especially in rural areas. Non-probability sampling also has higher error rates when compared with known benchmarks (e.g., Lehdonvirta et al. [2]). However, a recent study focusing on wildlife-related issues found that a non-probability Qualtrics sample out-performed a probability-based phone survey in terms of replicating benchmarks [3]. This study also showed that mail, phone, and internet-based surveys were all biased toward those who participated in wildlife-related activities (e.g., hunting, fishing), which likely drives greater interest in wildlife-related issues. Thus, it may not be unlikely that our data likewise over-represent people who have some interest in wildlife issues. However, that our panels do not show polarization or willingness to contact politicians give us confidence about the generalization of our results at the country population level. European nations being diverse in terms of their social and economic conditions, these conditions may affect the propensity of individuals to respond. For example, Daikeler et al. [4] showed that country-level response rates to web-based surveys tend to be higher (relative to traditional modes of contact; e.g., mailed survey) in countries with high population growth, high internet coverage, and lower (again relative to traditional modes) in countries with a high population age and high cell phone coverage. We mitigated for this possible bias by specifically requesting a quota of rural respondents (whether a respondent enters the category rural or urban is determined by Q9, with answers "Village" or "Isolated house" converting to rural, and answers "Large urban area" or "City" converting to urban). In a few countries, however, this targeted balance could not be fully achieved. For instance, rural respondents in Bulgaria, Croatia, and Romania were underrepresented relative to our targets. Nevertheless, this did not limit our statistical analyses, which were conducted within a Bayesian framework. In Germany, we applied a country-specific constraint to ensure that the respondent distribution reflected the population split between former East and West Germany [5], given the enduring political and cultural differences between these regions [6–8].

The survey was GDPR compliant. All participants provided informed consent prior to participation, and responses were anonymized by Qualtrics before being made available to the research team.

Survey questions

Q1: Large carnivores such as wolves, brown bears and lynx have been recolonizing parts of Europe in recent decades. Generally speaking, would you say that you support or oppose the recovery of large carnivores? (*Strongly oppose / Oppose / Neutral or not sure / Support / Strongly support*)

Q2: In my opinion, wolf populations in Europe should be… (*I don't know / Decreased greatly / Decreased / Stay about the same / Increased / Increased greatly*)

Q3: In my opinion, lynx populations in Europe should be… (*I don't know / Decreased greatly / Decreased / Stay about the same / Increased / Increased greatly*)

Q4: In my opinion, brown bear populations in Europe should be… (*I don't know / Decreased greatly / Decreased / Stay about the same / Increased / Increased greatly*)

Q5: Generally speaking, would you say that you support or oppose hunting large carnivores? (*Strongly oppose / Oppose / Neutral or not sure / Support / Strongly support*)

Q6: To what extent do you agree or disagree with the following:

1. If a bear attacks a person, that bear should be killed regardless of the circumstances.
2. Wolves that kill livestock should be killed.
3. A lynx that kills livestock should be killed.

(*Strongly agree / Somewhat agree / Neither agree nor disagree / Somewhat disagree / Strongly disagree*)

Q7: What is your sex? (*Male / Female*)

Q8: Enter the year you were born (in YYYY format). (note: data shows respondent's age)

Q9: How would you describe your current residence or community? (*Large urban area / City / Village / Isolated house*)

Q10: When it comes to politics, please indicate which of the following you consider yourself. (*Extreme left / Left (socio-democrat) / Centre / Right (conservative) / Extreme right*)

Q11: Below are a number of actions you could take in order to support the conservation of large carnivores in your country. Please indicate how likely or unlikely you are to... Write to or call a politician to express your support for carnivore restoration efforts. (*Very Unlikely / Somewhat Unlikely / Undecided / Somewhat Likely / Very Likely*)

Q12: Below are a number of actions you could take in order to oppose large carnivore populations in your country. Please indicate how likely or unlikely you are to... write to or call a politician to express your opposition to carnivore restoration efforts. (*Very Unlikely / Somewhat Unlikely / Undecided / Somewhat Likely / Very Likely*)

For Q11 and Q12, while the survey asked about several possible actions respondents could take, because our interest here is in policy, we report only those responses to the item asking about their willingness to contact a politician.

Survey translations in the country languages are available at the end of this document.

Supplementary Table 1.

Summary of survey responses by country (ISO 3166-1 alpha-2 code and name), showing the total number of respondents (N), and the number of female, male, rural, and urban respondents, as well as those younger and older than the country-specific median age.

	N	Female	Male	Rural	Urban	Young	Old
**AT** (Austria)	507	255	252	203	304	262	244
**BE** (Belgium)	510	255	255	255	255	227	283
**BG** (Bulgaria)	569	292	277	82	487	387	182
**CZ** (Czechia)	510	255	255	131	379	256	254
**DE** (Germany)	605	324	281	201	404	331	271
**DK** (Denmark)	511	255	256	256	255	238	270
**EE** (Estonia)	299	165	134	134	165	166	133
**ES** (Spain)	500	250	250	245	255	286	212
**FI** (Finland)	501	251	250	245	256	251	249
**FR** (France)	501	250	251	246	255	224	272
**GR** (Greece)	501	250	251	246	255	322	177
**HR** (Croatia)	327	168	159	82	245	203	124
**HU** (Hungary)	523	262	261	261	262	260	261
**IT** (Italy)	500	250	250	245	255	237	262
**LT** (Lithuania)	382	244	138	126	256	228	152
**LV** (Latvia)	110	55	55	28	82	68	42
**NL** (Netherlands)	501	264	237	245	256	217	282
**PL** (Poland)	500	250	250	244	256	255	241
**PT** (Portugal)	501	250	251	244	257	256	245
**RO** (Romania)	613	307	306	144	469	387	225
**SE** (Sweden)	500	250	250	245	255	239	259
**SI** (Slovenia)	335	178	157	155	180	177	157
**SK** (Slovakia)	501	251	250	245	256	255	245

Supplementary Table 2.
Median age (in 2021) [9] and percentage of the population living in rural areas [10] for each surveyed country.

	Median age	% rural
**AT**	43.6	39.3
**BE**	41.8	19.8
**BG**	46.4	33.4
**CZ**	43.6	38.8
**DE**	45.9	26.0
**DK**	42.2	32.5
**EE**	42.5	33.8
**ES**	44.6	17.1
**FI**	43.3	34.9
**FR**	42	35.7
**GR**	45.5	28.2
**HR**	45.3	41.8
**HU**	43.7	34.8
**IT**	47.6	23.5
**LT**	44.5	35.4
**LV**	43.9	35.6
**NL**	42.7	13.4
**PL**	41.9	38.7
**PT**	46.3	28.3
**RO**	43	44.4
**SE**	40.6	30.5
**SI**	44.4	48.7
**SK**	41.4	44.5

Supplementary Results

Supplementary Table 3.

Coefficients of the linear model for every country for the question "*Large carnivores such as wolves, brown bears and lynx have been recolonizing parts of Europe in recent decades. Generally speaking, would you say that you support or oppose the recovery of large carnivores?*" (Q1), median values with 95% credible intervals (shown with a beige background when 0 is not included in the interval). All variables are categorical except age. Intercepts are rural, female and political center.

Countries	Rural – Urban	Female – Male	Age	Extreme left	Left	Right	Extreme right
**AT**	0.29 (0.08, 0.5)	0.26 (0.05, 0.47)	0 (-0.01, 0)	1.11 (-0.1, 2.4)	0.18 (-0.08, 0.44)	-0.28 (-0.55, 0)	0.07 (-0.7, 0.85)
**BE**	0.02 (-0.17, 0.21)	0.05 (-0.13, 0.24)	0 (0, 0.01)	0.48 (-0.02, 0.98)	0.16 (-0.1, 0.41)	-0.07 (-0.3, 0.15)	0.34 (-0.15, 0.84)
**BG**	0.12 (-0.16, 0.4)	0.36 (0.17, 0.56)	0 (-0.01, 0.01)	-0.1 (-0.65, 0.45)	-0.13 (-0.4, 0.14)	-0.01 (-0.28, 0.27)	-0.02 (-0.54, 0.5)
**CZ**	0.08 (-0.09, 0.26)	0.02 (-0.14, 0.17)	-0.01 (-0.02, -0.01)	0.09 (-0.49, 0.66)	-0.18 (-0.41, 0.04)	0.07 (-0.13, 0.28)	0.5 (-0.08, 1.1)
**DE**	0.04 (-0.18, 0.25)	-0.05 (-0.25, 0.15)	-0.01 (-0.01, 0)	0.06 (-0.47, 0.59)	0.34 (0.09, 0.58)	-0.07 (-0.34, 0.2)	-0.22 (-1.32, 0.86)
**DK**	-0.22 (-0.45, 0.02)	-0.04 (-0.28, 0.2)	-0.01 (-0.01, 0)	1.08 (0.67, 1.5)	0.01 (-0.29, 0.31)	0.11 (-0.21, 0.44)	-0.48 (-1.06, 0.1)
**EE**	0.28 (0.05, 0.51)	0.23 (-0.01, 0.46)	0 (-0.01, 0.01)	0.86 (0.03, 1.73)	0.1 (-0.22, 0.41)	0.08 (-0.19, 0.35)	0.69 (-0.44, 1.86)
**ES**	-0.03 (-0.26, 0.19)	0.15 (-0.09, 0.38)	0.01 (0, 0.02)	0.14 (-0.33, 0.62)	0.03 (-0.24, 0.3)	-0.11 (-0.42, 0.19)	0.18 (-0.57, 0.94)
**FI**	0.23 (0.04, 0.43)	0.12 (-0.07, 0.31)	0 (-0.01, 0.01)	-0.18 (-0.7, 0.34)	0.15 (-0.09, 0.4)	-0.18 (-0.42, 0.06)	0.44 (-0.07, 0.96)
**FR**	0.1 (-0.1, 0.3)	0.17 (-0.03, 0.37)	-0.01 (-0.01, 0)	0.4 (-0.11, 0.91)	0.31 (0.06, 0.56)	0 (-0.28, 0.29)	0.13 (-0.19, 0.45)
**GR**	0.01 (-0.17, 0.2)	0.07 (-0.11, 0.26)	0.01 (0, 0.02)	0.07 (-0.48, 0.62)	0.28 (0.04, 0.52)	0.05 (-0.18, 0.28)	-0.49 (-0.92, -0.06)
**HR**	0.06 (-0.23, 0.35)	-0.07 (-0.32, 0.18)	0.01 (0, 0.02)	0.37 (-0.5, 1.28)	-0.08 (-0.37, 0.2)	-0.33 (-0.69, 0.01)	-0.06 (-0.78, 0.67)
**HU**	0.17 (-0.05, 0.4)	0.02 (-0.21, 0.24)	0 (-0.01, 0.01)	-0.54 (-1.29, 0.2)	0.25 (-0.04, 0.54)	-0.1 (-0.37, 0.17)	0.05 (-0.46, 0.57)
**IT**	0.12 (-0.09, 0.34)	0.34 (0.14, 0.54)	0 (0, 0.01)	0.38 (-0.05, 0.82)	0.03 (-0.22, 0.29)	-0.07 (-0.33, 0.19)	-0.29 (-0.88, 0.3)
**LT**	0.07 (-0.17, 0.32)	0.07 (-0.17, 0.3)	-0.01 (-0.02, 0)	0.01 (-0.53, 0.55)	-0.16 (-0.43, 0.11)	0.16 (-0.14, 0.46)	0.37 (-0.31, 1.05)
**LV**	0.23 (-0.2, 0.66)	-0.07 (-0.5, 0.35)	0 (-0.02, 0.02)	0.44 (-0.68, 1.59)	0.47 (-0.03, 0.99)	0.21 (-0.26, 0.7)	1.14 (0.13, 2.16)
**NL**	0.14 (-0.06, 0.35)	0.04 (-0.16, 0.24)	-0.01 (-0.02, -0.01)	0.29 (-0.38, 0.96)	0.47 (0.23, 0.72)	-0.15 (-0.4, 0.1)	0.39 (-0.26, 1.04)
**PL**	0.24 (0.05, 0.43)	0.1 (-0.09, 0.29)	0 (0, 0.01)	0.44 (0.04, 0.85)	0.06 (-0.18, 0.31)	-0.02 (-0.27, 0.23)	0.31 (-0.19, 0.82)
**PT**	-0.19 (-0.43, 0.04)	0.11 (-0.11, 0.34)	0 (-0.01, 0.01)	0.4 (-0.22, 1.01)	0.32 (0.05, 0.58)	0.02 (-0.28, 0.32)	-0.13 (-0.73, 0.47)
**RO**	0.29 (0.07, 0.5)	0.01 (-0.16, 0.19)	0 (0, 0.01)	-0.34 (-0.83, 0.14)	0.01 (-0.21, 0.22)	0.14 (-0.09, 0.39)	-0.01 (-0.52, 0.5)
**SE**	0.04 (-0.18, 0.27)	-0.03 (-0.26, 0.19)	0.01 (0, 0.02)	0.75 (0.25, 1.27)	0.05 (-0.23, 0.33)	-0.12 (-0.43, 0.18)	0.25 (-0.24, 0.74)
**SI**	0.15 (-0.09, 0.4)	0.12 (-0.12, 0.37)	-0.01 (-0.02, 0)	0.42 (-0.21, 1.06)	0.24 (-0.05, 0.53)	-0.32 (-0.76, 0.12)	-0.97 (-1.81, -0.13)
**SK**	0.2 (0.02, 0.38)	0.18 (0, 0.37)	-0.01 (-0.02, -0.01)	0.46 (-0.37, 1.29)	0.21 (-0.03, 0.45)	0.08 (-0.17, 0.34)	-0.36 (-1.16, 0.43)

Supplementary Table 4.

Coefficients of the linear model for every country for the question "*In my opinion, wolf populations in Europe should be…*" (Q2), median values with 95% credible intervals (shown with a beige background when 0 is not included in the interval). All variables are categorical except age. Intercepts are rural, female and political center.

Countries	Rural – Urban	Female – Male	Age	Extreme left	Left	Right	Extreme right
**AT**	0.14 (-0.05, 0.33)	0.1 (-0.09, 0.29)	-0.01 (-0.02, -0.01)	0.49 (-0.54, 1.51)	0.24 (0.01, 0.48)	-0.18 (-0.43, 0.08)	0.26 (-0.47, 1)
**BE**	0.06 (-0.13, 0.25)	-0.08 (-0.27, 0.12)	-0.01 (-0.02, -0.01)	0.48 (-0.03, 0.99)	0.18 (-0.08, 0.45)	-0.11 (-0.34, 0.12)	-0.05 (-0.54, 0.43)
**BG**	0.23 (-0.04, 0.5)	0 (-0.2, 0.19)	-0.01 (-0.02, -0.01)	-0.35 (-0.85, 0.15)	0.02 (-0.24, 0.29)	-0.07 (-0.34, 0.2)	0.01 (-0.49, 0.5)
**CZ**	0.02 (-0.16, 0.21)	0.1 (-0.07, 0.26)	-0.02 (-0.03, -0.02)	-0.05 (-0.7, 0.58)	-0.24 (-0.48, 0.01)	0.12 (-0.09, 0.34)	0.5 (-0.1, 1.11)
**DE**	0.02 (-0.17, 0.2)	-0.1 (-0.27, 0.08)	-0.02 (-0.03, -0.01)	0.31 (-0.13, 0.75)	0.41 (0.19, 0.63)	0.1 (-0.14, 0.34)	-0.14 (-1.16, 0.88)
**DK**	-0.08 (-0.29, 0.14)	0.13 (-0.09, 0.34)	-0.02 (-0.02, -0.01)	0.91 (0.55, 1.28)	0.15 (-0.13, 0.41)	0.01 (-0.28, 0.31)	-0.03 (-0.53, 0.47)
**EE**	0.35 (0.11, 0.58)	0.21 (-0.03, 0.45)	0 (-0.01, 0)	0.15 (-0.7, 1.01)	0.21 (-0.12, 0.54)	-0.01 (-0.28, 0.27)	-0.05 (-1.14, 1.03)
**ES**	-0.09 (-0.3, 0.12)	-0.06 (-0.27, 0.16)	-0.01 (-0.02, 0)	0.07 (-0.38, 0.52)	0.14 (-0.11, 0.39)	-0.1 (-0.38, 0.19)	-0.15 (-0.86, 0.57)
**FI**	0.1 (-0.08, 0.3)	0.1 (-0.08, 0.28)	-0.01 (-0.01, 0)	-0.61 (-1.15, -0.07)	0.04 (-0.21, 0.28)	-0.4 (-0.63, -0.16)	0.13 (-0.36, 0.62)
**FR**	0.14 (-0.06, 0.34)	0.03 (-0.17, 0.22)	-0.01 (-0.02, -0.01)	0.37 (-0.11, 0.85)	0 (-0.25, 0.25)	-0.2 (-0.48, 0.08)	-0.2 (-0.52, 0.12)
**GR**	0.21 (0, 0.43)	-0.06 (-0.27, 0.16)	-0.01 (-0.02, 0)	-0.05 (-0.74, 0.65)	0.06 (-0.22, 0.35)	0.01 (-0.26, 0.28)	0.46 (-0.03, 0.94)
**HR**	0.09 (-0.16, 0.34)	-0.03 (-0.24, 0.18)	-0.01 (-0.02, 0)	0.02 (-0.66, 0.7)	0.01 (-0.23, 0.25)	-0.35 (-0.65, -0.05)	-0.48 (-1.07, 0.1)
**HU**	0.1 (-0.08, 0.28)	-0.1 (-0.28, 0.08)	-0.01 (-0.02, 0)	-0.42 (-1.06, 0.21)	-0.03 (-0.26, 0.2)	-0.25 (-0.47, -0.03)	-0.04 (-0.44, 0.35)
**IT**	0.1 (-0.09, 0.3)	0.14 (-0.04, 0.32)	-0.01 (-0.02, 0)	0.5 (0.13, 0.87)	0.04 (-0.19, 0.26)	-0.17 (-0.4, 0.05)	-0.72 (-1.25, -0.2)
**LT**	0.11 (-0.12, 0.33)	0.25 (0.04, 0.47)	-0.02 (-0.03, -0.01)	-0.08 (-0.59, 0.44)	-0.08 (-0.33, 0.16)	0.03 (-0.24, 0.31)	-0.63 (-1.27, 0)
**LV**	-0.04 (-0.48, 0.41)	-0.24 (-0.72, 0.24)	0 (-0.02, 0.02)	0.23 (-0.99, 1.46)	0.21 (-0.32, 0.75)	0.17 (-0.35, 0.69)	0.31 (-1.06, 1.69)
**NL**	0.22 (0.01, 0.44)	0.08 (-0.14, 0.3)	-0.02 (-0.03, -0.02)	1.29 (0.46, 2.13)	0.29 (0.03, 0.55)	-0.32 (-0.58, -0.05)	-0.31 (-1.06, 0.43)
**PL**	0.15 (-0.05, 0.34)	0 (-0.19, 0.19)	-0.02 (-0.02, -0.01)	0.09 (-0.31, 0.49)	0.24 (-0.02, 0.5)	-0.13 (-0.39, 0.13)	-0.27 (-0.81, 0.27)
**PT**	-0.02 (-0.21, 0.17)	-0.12 (-0.3, 0.06)	-0.01 (-0.02, -0.01)	0.31 (-0.19, 0.82)	0.16 (-0.05, 0.37)	-0.09 (-0.34, 0.16)	-0.09 (-0.56, 0.37)
**RO**	0.12 (-0.09, 0.33)	0.11 (-0.07, 0.28)	-0.01 (-0.02, 0)	-0.49 (-0.94, -0.05)	0 (-0.21, 0.21)	0.2 (-0.03, 0.43)	0.36 (-0.13, 0.85)
**SE**	0.3 (0.1, 0.5)	0.18 (-0.02, 0.38)	-0.01 (-0.01, 0)	0.28 (-0.16, 0.72)	0.11 (-0.14, 0.36)	-0.06 (-0.33, 0.21)	-0.16 (-0.59, 0.28)
**SI**	0.12 (-0.1, 0.33)	0.11 (-0.11, 0.32)	-0.02 (-0.03, -0.01)	0.47 (-0.08, 1.01)	0.28 (0.03, 0.53)	-0.52 (-0.9, -0.15)	-1.21 (-1.95, -0.49)
**SK**	0.15 (-0.05, 0.35)	0.25 (0.04, 0.45)	-0.02 (-0.02, -0.01)	-0.56 (-1.41, 0.3)	-0.05 (-0.32, 0.2)	0.08 (-0.2, 0.36)	-0.6 (-1.67, 0.47)

Supplementary Table 5.

Coefficients of the linear model for every country for the question "*In my opinion, lynx populations in Europe should be…*" (Q3), median values with 95% credible intervals (shown with a beige background when 0 is not included in the interval). All variables are categorical except age. Intercepts are rural, female and political center.

Countries	Rural – Urban	Female – Male	Age	Extreme left	Left	Right	Extreme right
AT	-0.04 (-0.24, 0.16)	0.21 (0.01, 0.41)	0 (-0.01, 0)	0.58 (-0.48, 1.64)	0.38 (0.13, 0.62)	0.15 (-0.11, 0.42)	0.78 (0.01, 1.58)
BE	0.07 (-0.13, 0.27)	0.05 (-0.15, 0.25)	-0.01 (-0.02, -0.01)	0.41 (-0.11, 0.94)	0.33 (0.05, 0.61)	-0.05 (-0.29, 0.2)	-0.14 (-0.65, 0.37)
BG	0.31 (0.04, 0.58)	0.11 (-0.09, 0.3)	-0.02 (-0.02, -0.01)	-0.3 (-0.78, 0.2)	0.05 (-0.21, 0.32)	0.16 (-0.1, 0.43)	-0.18 (-0.67, 0.31)
CZ	-0.07 (-0.28, 0.14)	0.2 (0.02, 0.38)	-0.01 (-0.01, 0)	-0.36 (-1.1, 0.39)	-0.17 (-0.44, 0.09)	-0.04 (-0.28, 0.2)	-0.02 (-0.67, 0.64)
DE	-0.21 (-0.4, -0.02)	0.04 (-0.13, 0.23)	-0.01 (-0.02, 0)	0.39 (-0.06, 0.85)	0.33 (0.11, 0.56)	0.01 (-0.24, 0.26)	0.06 (-0.87, 0.98)
DK	-0.07 (-0.28, 0.13)	0.21 (0.01, 0.42)	-0.01 (-0.02, 0)	0.65 (0.3, 1)	0.12 (-0.14, 0.38)	0.01 (-0.28, 0.29)	-0.42 (-0.9, 0.07)
EE	0.35 (0.09, 0.61)	0.34 (0.08, 0.6)	-0.01 (-0.02, 0)	-0.03 (-0.86, 0.81)	0.06 (-0.29, 0.41)	-0.08 (-0.38, 0.22)	-0.26 (-1.43, 0.89)
ES	0.09 (-0.14, 0.32)	0.02 (-0.22, 0.25)	0 (-0.01, 0)	0.08 (-0.39, 0.55)	0.07 (-0.2, 0.35)	-0.05 (-0.36, 0.26)	-0.17 (-0.93, 0.6)
FI	0.07 (-0.12, 0.26)	0.13 (-0.06, 0.31)	0 (-0.01, 0)	-0.38 (-0.91, 0.16)	0.13 (-0.12, 0.37)	-0.09 (-0.32, 0.15)	0.45 (-0.05, 0.94)
FR	0.17 (-0.02, 0.37)	0.26 (0.06, 0.46)	-0.01 (-0.02, -0.01)	0.11 (-0.37, 0.6)	0.01 (-0.24, 0.26)	-0.16 (-0.43, 0.12)	-0.22 (-0.53, 0.09)
GR	0.14 (-0.07, 0.36)	0.01 (-0.2, 0.23)	0 (-0.01, 0.01)	0.61 (-0.05, 1.27)	0.15 (-0.13, 0.43)	-0.14 (-0.41, 0.13)	0.09 (-0.39, 0.58)
HR	0.24 (-0.02, 0.5)	0.21 (-0.01, 0.43)	0 (-0.01, 0)	0.49 (-0.25, 1.24)	0.16 (-0.1, 0.41)	-0.37 (-0.68, -0.06)	-0.1 (-0.72, 0.51)
HU	0.06 (-0.13, 0.24)	0.07 (-0.12, 0.25)	-0.01 (-0.01, 0)	-0.44 (-1.1, 0.22)	0.02 (-0.22, 0.26)	-0.14 (-0.36, 0.09)	0.25 (-0.18, 0.67)
IT	0.11 (-0.09, 0.31)	0.14 (-0.04, 0.32)	0 (-0.01, 0)	0.55 (0.17, 0.95)	0.08 (-0.15, 0.32)	-0.21 (-0.45, 0.02)	-0.26 (-0.78, 0.26)
LT	0.04 (-0.2, 0.28)	0.26 (0.03, 0.49)	0 (-0.01, 0)	-0.25 (-0.81, 0.31)	-0.1 (-0.38, 0.16)	0.01 (-0.29, 0.32)	0.12 (-0.53, 0.77)
LV	0.05 (-0.33, 0.45)	0.22 (-0.2, 0.65)	-0.01 (-0.02, 0.01)	-0.28 (-1.44, 0.89)	0.43 (-0.03, 0.92)	0.57 (0.11, 1.06)	0.72 (-0.26, 1.75)
NL	0.27 (0.06, 0.48)	0.1 (-0.11, 0.31)	-0.02 (-0.03, -0.01)	1.21 (0.42, 2.02)	0.48 (0.22, 0.74)	-0.23 (-0.49, 0.03)	-0.21 (-0.93, 0.49)
PL	-0.05 (-0.26, 0.16)	-0.04 (-0.25, 0.18)	0 (-0.01, 0)	0.21 (-0.23, 0.67)	0.38 (0.1, 0.65)	-0.03 (-0.31, 0.24)	-0.04 (-0.64, 0.55)
PT	0.03 (-0.17, 0.24)	0.07 (-0.13, 0.27)	-0.01 (-0.02, 0)	-0.26 (-0.78, 0.27)	0.02 (-0.21, 0.25)	-0.17 (-0.43, 0.1)	-0.2 (-0.7, 0.3)
RO	0.18 (-0.02, 0.38)	0.07 (-0.1, 0.23)	0 (-0.01, 0)	-0.29 (-0.74, 0.16)	-0.09 (-0.29, 0.12)	0.11 (-0.12, 0.33)	-0.08 (-0.56, 0.4)
SE	0.05 (-0.16, 0.26)	0.21 (0, 0.42)	0 (-0.01, 0)	0.18 (-0.27, 0.64)	0.14 (-0.12, 0.4)	-0.05 (-0.32, 0.23)	0.05 (-0.4, 0.49)
SI	-0.07 (-0.3, 0.16)	0.3 (0.06, 0.53)	-0.01 (-0.02, 0)	0.24 (-0.32, 0.82)	0.47 (0.2, 0.74)	-0.27 (-0.67, 0.15)	-1.08 (-1.83, -0.32)
SK	0.21 (0, 0.42)	0.35 (0.13, 0.56)	-0.01 (-0.02, 0)	0.01 (-0.91, 0.96)	-0.06 (-0.33, 0.21)	0.13 (-0.15, 0.42)	-0.6 (-1.7, 0.52)

Supplementary Table 6.

Coefficients of the linear model for every country for the question "*In my opinion, brown bear populations in Europe should be…*" (Q4), median values with 95% credible intervals (shown with a beige background when 0 is not included in the interval). All variables are categorical except age. Intercepts are rural, female and political center.

Countries	Rural – Urban	Female – Male	Age	Extreme left	Left	Right	Extreme right
**AT**	0.14 (-0.05, 0.32)	0.07 (-0.11, 0.26)	-0.01 (-0.02, 0)	0.49 (-0.49, 1.47)	0.27 (0.05, 0.49)	-0.02 (-0.26, 0.23)	1.01 (0.29, 1.73)
**BE**	0.04 (-0.16, 0.24)	0.03 (-0.17, 0.22)	-0.01 (-0.01, 0)	0.17 (-0.36, 0.7)	0.15 (-0.12, 0.43)	-0.1 (-0.34, 0.14)	-0.05 (-0.56, 0.47)
**BG**	0.38 (0.12, 0.64)	0.02 (-0.16, 0.21)	-0.01 (-0.02, 0)	-0.22 (-0.7, 0.27)	0.04 (-0.21, 0.29)	0.08 (-0.17, 0.34)	-0.06 (-0.54, 0.42)
**CZ**	-0.04 (-0.22, 0.15)	0.11 (-0.05, 0.27)	-0.02 (-0.02, -0.01)	0.24 (-0.38, 0.85)	-0.04 (-0.27, 0.2)	0.11 (-0.1, 0.32)	0.26 (-0.32, 0.84)
**DE**	0 (-0.18, 0.19)	-0.11 (-0.28, 0.07)	-0.02 (-0.02, -0.01)	0.57 (0.13, 1.01)	0.35 (0.13, 0.57)	0.06 (-0.18, 0.3)	0.41 (-0.61, 1.45)
**DK**	-0.01 (-0.2, 0.18)	0.03 (-0.16, 0.22)	-0.01 (-0.01, 0)	0.8 (0.49, 1.12)	0.12 (-0.12, 0.36)	0.27 (0.02, 0.53)	-0.1 (-0.55, 0.35)
**EE**	0.37 (0.14, 0.61)	0.23 (-0.01, 0.47)	0 (-0.01, 0.01)	0.02 (-0.75, 0.78)	0.03 (-0.29, 0.36)	-0.08 (-0.35, 0.19)	0.83 (-0.54, 2.27)
**ES**	0.05 (-0.16, 0.27)	0.04 (-0.18, 0.25)	-0.01 (-0.01, 0)	0.06 (-0.37, 0.5)	0.08 (-0.18, 0.33)	-0.04 (-0.33, 0.25)	-0.4 (-1.12, 0.32)
**FI**	0.02 (-0.17, 0.21)	0.01 (-0.17, 0.2)	0 (-0.01, 0)	-0.37 (-0.9, 0.16)	0.12 (-0.12, 0.36)	-0.14 (-0.37, 0.1)	0.57 (0.08, 1.06)
**FR**	0.15 (-0.04, 0.35)	0.27 (0.08, 0.47)	-0.02 (-0.02, -0.01)	0.36 (-0.11, 0.84)	0.1 (-0.14, 0.35)	0.03 (-0.25, 0.31)	0.08 (-0.23, 0.39)
**GR**	0.17 (-0.04, 0.37)	0 (-0.21, 0.21)	0 (-0.01, 0.01)	0.55 (-0.1, 1.2)	0.06 (-0.21, 0.34)	-0.03 (-0.29, 0.22)	0.33 (-0.15, 0.83)
**HR**	0.04 (-0.2, 0.28)	-0.04 (-0.25, 0.17)	-0.01 (-0.01, 0)	0.51 (-0.25, 1.29)	-0.11 (-0.35, 0.13)	-0.39 (-0.69, -0.09)	-0.6 (-1.22, 0.02)
**HU**	0.14 (-0.04, 0.31)	-0.08 (-0.26, 0.1)	-0.01 (-0.02, -0.01)	-0.28 (-0.9, 0.33)	0.09 (-0.13, 0.33)	-0.26 (-0.49, -0.04)	-0.12 (-0.52, 0.27)
**IT**	0.1 (-0.09, 0.29)	0.1 (-0.07, 0.28)	-0.01 (-0.02, 0)	0.45 (0.09, 0.82)	0.03 (-0.2, 0.25)	-0.18 (-0.4, 0.05)	-0.15 (-0.67, 0.36)
**LT**	0.23 (-0.01, 0.46)	0.07 (-0.16, 0.3)	-0.01 (-0.02, 0)	-0.35 (-0.89, 0.18)	-0.05 (-0.31, 0.22)	0.02 (-0.28, 0.31)	0.14 (-0.52, 0.8)
**LV**	-0.06 (-0.5, 0.38)	-0.12 (-0.59, 0.34)	0 (-0.01, 0.02)	0.63 (-0.66, 1.93)	0.44 (-0.07, 0.96)	0.37 (-0.14, 0.87)	0.03 (-1.11, 1.18)
**NL**	0.18 (-0.03, 0.39)	0.17 (-0.04, 0.38)	-0.02 (-0.03, -0.02)	0.74 (-0.09, 1.57)	0.43 (0.17, 0.69)	-0.2 (-0.46, 0.06)	-0.27 (-0.97, 0.43)
**PL**	0.04 (-0.15, 0.23)	-0.07 (-0.25, 0.12)	0 (-0.01, 0)	0.12 (-0.27, 0.5)	0.24 (-0.01, 0.48)	-0.06 (-0.31, 0.18)	0.18 (-0.37, 0.74)
**PT**	-0.03 (-0.22, 0.16)	-0.1 (-0.28, 0.08)	-0.01 (-0.02, 0)	0.18 (-0.31, 0.66)	0.07 (-0.14, 0.28)	-0.1 (-0.35, 0.15)	0.1 (-0.35, 0.55)
**RO**	-0.04 (-0.26, 0.18)	0.01 (-0.17, 0.19)	-0.02 (-0.03, -0.01)	-0.51 (-1.01, -0.02)	-0.09 (-0.32, 0.13)	0.02 (-0.22, 0.26)	0.38 (-0.12, 0.9)
**SE**	0.14 (-0.06, 0.34)	0.09 (-0.1, 0.29)	0 (-0.01, 0)	0.43 (-0.01, 0.87)	0.19 (-0.06, 0.43)	-0.05 (-0.31, 0.21)	0.17 (-0.26, 0.59)
**SI**	0.23 (0.02, 0.44)	0.18 (-0.03, 0.39)	-0.02 (-0.03, -0.01)	0.24 (-0.28, 0.76)	0.07 (-0.18, 0.31)	-0.4 (-0.77, -0.02)	-0.95 (-1.66, -0.25)
**SK**	0.17 (-0.04, 0.38)	0.17 (-0.04, 0.39)	-0.02 (-0.03, -0.01)	-1.26 (-2.28, -0.28)	0.05 (-0.22, 0.33)	-0.02 (-0.32, 0.27)	-0.34 (-1.46, 0.8)

Supplementary Table 7.

Coefficients of the linear model for every country for the question "*Generally speaking, would you say that you support or oppose hunting large carnivores?*" (Q5), median values with 95% credible intervals (shown with a beige background when 0 is not included in the interval). All variables are categorical except age. Intercepts are rural, female and political center.

Countries	Rural – Urban	Female – Male	Age	Extreme left	Left	Right	Extreme right
**AT**	-0.21 (-0.43, 0.01)	0.12 (-0.11, 0.34)	0 (-0.01, 0.01)	-0.17 (-1.43, 1.03)	-0.07 (-0.35, 0.21)	0.09 (-0.2, 0.39)	-0.19 (-1.02, 0.64)
**BE**	-0.06 (-0.26, 0.14)	0.38 (0.18, 0.58)	-0.01 (-0.01, 0)	-0.46 (-1.01, 0.08)	-0.38 (-0.65, -0.11)	0.12 (-0.12, 0.36)	-0.04 (-0.57, 0.48)
**BG**	-0.32 (-0.61, -0.05)	0.31 (0.11, 0.51)	0 (0, 0.01)	-0.26 (-0.81, 0.29)	-0.16 (-0.43, 0.11)	-0.18 (-0.46, 0.09)	0.11 (-0.41, 0.64)
**CZ**	-0.11 (-0.34, 0.11)	0.26 (0.06, 0.47)	0.01 (0.01, 0.02)	-0.07 (-0.84, 0.69)	0.04 (-0.24, 0.33)	-0.22 (-0.49, 0.04)	0.23 (-0.49, 0.97)
**DE**	-0.04 (-0.25, 0.18)	0.27 (0.07, 0.48)	0.01 (0, 0.01)	0.18 (-0.36, 0.72)	-0.33 (-0.59, -0.07)	0.11 (-0.16, 0.4)	0.48 (-0.64, 1.59)
**DK**	-0.07 (-0.3, 0.16)	0.26 (0.04, 0.49)	0 (0, 0.01)	-0.5 (-0.89, -0.11)	0.17 (-0.12, 0.46)	0.09 (-0.22, 0.4)	0.47 (-0.08, 1.02)
**EE**	-0.3 (-0.53, -0.08)	0.1 (-0.13, 0.33)	0.01 (0, 0.02)	0.34 (-0.45, 1.13)	-0.19 (-0.5, 0.12)	0.22 (-0.05, 0.48)	-0.65 (-1.75, 0.43)
**ES**	-0.13 (-0.36, 0.1)	0.31 (0.07, 0.55)	0 (-0.01, 0.01)	-0.4 (-0.89, 0.08)	-0.27 (-0.56, 0)	0.34 (0.03, 0.65)	0.63 (-0.12, 1.39)
**FI**	-0.27 (-0.47, -0.07)	0.15 (-0.04, 0.34)	0 (-0.01, 0.01)	-0.3 (-0.83, 0.21)	-0.24 (-0.49, 0.01)	0.25 (0.01, 0.5)	-0.13 (-0.66, 0.38)
**FR**	-0.15 (-0.37, 0.07)	0.29 (0.07, 0.5)	0 (-0.01, 0.01)	-0.19 (-0.72, 0.34)	-0.2 (-0.47, 0.07)	0.03 (-0.27, 0.34)	0.07 (-0.28, 0.41)
**GR**	-0.11 (-0.29, 0.08)	0.29 (0.1, 0.48)	-0.01 (-0.02, 0)	-0.35 (-0.94, 0.24)	-0.47 (-0.72, -0.21)	-0.11 (-0.35, 0.13)	0.5 (0.07, 0.94)
**HR**	-0.11 (-0.37, 0.13)	0.24 (0.02, 0.46)	-0.01 (-0.01, 0)	-0.43 (-1.22, 0.32)	-0.06 (-0.3, 0.19)	0.46 (0.16, 0.77)	0.25 (-0.37, 0.88)
**HU**	-0.21 (-0.41, -0.02)	0.36 (0.16, 0.56)	0 (0, 0.01)	-0.21 (-0.88, 0.46)	-0.08 (-0.34, 0.17)	0.3 (0.06, 0.54)	0.05 (-0.39, 0.5)
**IT**	-0.01 (-0.24, 0.23)	0.1 (-0.12, 0.32)	0 (-0.01, 0.01)	-0.59 (-1.08, -0.11)	-0.15 (-0.42, 0.13)	0.24 (-0.04, 0.51)	0.68 (0.05, 1.32)
**LT**	-0.03 (-0.25, 0.19)	0.28 (0.06, 0.5)	0.02 (0.01, 0.03)	-0.22 (-0.73, 0.28)	0.07 (-0.19, 0.33)	0.04 (-0.24, 0.32)	-0.26 (-0.9, 0.38)
**LV**	0.25 (-0.15, 0.66)	0.57 (0.16, 0.98)	-0.01 (-0.03, 0.01)	-0.97 (-2.21, 0.19)	-0.21 (-0.69, 0.26)	0.12 (-0.33, 0.57)	-0.28 (-1.22, 0.66)
**NL**	-0.1 (-0.3, 0.1)	0.13 (-0.07, 0.33)	0.01 (0, 0.01)	-0.36 (-1.04, 0.31)	-0.4 (-0.65, -0.16)	0.1 (-0.14, 0.36)	0.22 (-0.41, 0.87)
**PL**	-0.19 (-0.38, 0)	0.29 (0.1, 0.48)	0 (-0.01, 0.01)	-0.47 (-0.9, -0.04)	-0.24 (-0.49, 0.02)	0.26 (0.01, 0.52)	0.12 (-0.39, 0.63)
**PT**	0.19 (-0.04, 0.43)	0.3 (0.08, 0.53)	0 (-0.01, 0.01)	-0.36 (-1, 0.25)	-0.12 (-0.39, 0.14)	0.08 (-0.22, 0.37)	-0.03 (-0.62, 0.56)
**RO**	-0.12 (-0.34, 0.1)	0.17 (-0.01, 0.36)	0 (0, 0.01)	-0.08 (-0.58, 0.42)	0.04 (-0.18, 0.27)	0.03 (-0.22, 0.28)	-0.17 (-0.69, 0.35)
**SE**	-0.1 (-0.31, 0.11)	0.3 (0.09, 0.52)	0 (0, 0.01)	-0.82 (-1.3, -0.34)	-0.36 (-0.63, -0.1)	0.15 (-0.14, 0.43)	0.04 (-0.44, 0.52)
**SI**	-0.38 (-0.61, -0.16)	0.23 (0.01, 0.46)	0.01 (0, 0.02)	-0.66 (-1.26, -0.07)	-0.47 (-0.73, -0.21)	0.29 (-0.11, 0.69)	0.73 (-0.02, 1.49)
**SK**	-0.11 (-0.3, 0.07)	0.11 (-0.08, 0.3)	0.01 (0, 0.02)	1.12 (0.26, 1.99)	-0.13 (-0.37, 0.12)	0.18 (-0.08, 0.45)	0.42 (-0.44, 1.27)

Supplementary Table 8.

Coefficients of the linear model for every country for the statement “*If a bear attacks a person, that bear should be killed regardless of the circumstances*” (Q6.1), median values with 95% credible intervals (shown with a beige background when 0 is not included in the interval). All variables are categorical except age. Intercepts are rural, female and political center.

Countries	Rural – Urban	Female – Male	Age	Extreme left	Left	Right	Extreme right
**AT**	-0.05 (-0.29, 0.2)	0.15 (-0.09, 0.4)	0.01 (0, 0.02)	-0.18 (-1.52, 1.15)	-0.22 (-0.52, 0.08)	0.15 (-0.17, 0.48)	0.18 (-0.72, 1.08)
**BE**	0.27 (0.02, 0.51)	0.27 (0.02, 0.51)	0.01 (0, 0.01)	0.05 (-0.6, 0.71)	-0.2 (-0.54, 0.13)	0.46 (0.16, 0.76)	0.1 (-0.55, 0.75)
**BG**	-0.35 (-0.75, 0.05)	0.32 (0.04, 0.6)	0.02 (0.01, 0.03)	0.83 (0.04, 1.61)	0.39 (0, 0.78)	0.26 (-0.12, 0.65)	0.89 (0.14, 1.63)
**CZ**	0.06 (-0.15, 0.27)	0.19 (0, 0.38)	0.01 (0.01, 0.02)	0.24 (-0.43, 0.92)	0.41 (0.15, 0.69)	-0.05 (-0.3, 0.19)	0.53 (-0.15, 1.23)
**DE**	-0.03 (-0.3, 0.23)	0.25 (0, 0.5)	0.01 (0, 0.02)	0.08 (-0.57, 0.73)	-0.23 (-0.53, 0.08)	0.12 (-0.22, 0.46)	0.89 (-0.51, 2.32)
**DK**	0.05 (-0.22, 0.32)	0.14 (-0.14, 0.41)	0.01 (0.01, 0.02)	-0.98 (-1.44, -0.53)	0.14 (-0.2, 0.49)	0.02 (-0.35, 0.4)	1.18 (0.49, 1.89)
**EE**	-0.14 (-0.5, 0.23)	0.21 (-0.16, 0.59)	0.01 (0, 0.03)	0.68 (-0.6, 1.98)	0.02 (-0.49, 0.52)	0.45 (0.03, 0.89)	0.84 (-0.92, 2.63)
**ES**	0.1 (-0.2, 0.39)	0.47 (0.17, 0.78)	0.01 (0, 0.03)	-0.33 (-0.95, 0.29)	-0.27 (-0.64, 0.09)	0.07 (-0.33, 0.47)	-0.05 (-1.08, 0.95)
**FI**	0.01 (-0.29, 0.31)	0.16 (-0.13, 0.45)	0.01 (0, 0.02)	0.29 (-0.5, 1.08)	-0.35 (-0.72, 0.02)	0.18 (-0.19, 0.55)	-0.02 (-0.82, 0.78)
**FR**	-0.06 (-0.31, 0.18)	0.16 (-0.08, 0.4)	0.01 (0, 0.02)	-0.07 (-0.66, 0.53)	-0.15 (-0.45, 0.15)	-0.08 (-0.42, 0.26)	0.05 (-0.34, 0.43)
**GR**	0.04 (-0.22, 0.29)	0.31 (0.06, 0.58)	0.01 (0, 0.02)	-0.29 (-1.09, 0.5)	-0.15 (-0.49, 0.19)	-0.02 (-0.35, 0.3)	0.69 (0.09, 1.29)
**HR**	-0.19 (-0.57, 0.18)	0.29 (-0.03, 0.61)	0 (-0.01, 0.01)	-0.39 (-1.56, 0.75)	-0.15 (-0.52, 0.22)	0.3 (-0.15, 0.76)	0.05 (-0.9, 1)
**HU**	-0.12 (-0.4, 0.17)	-0.06 (-0.35, 0.23)	0.01 (0, 0.02)	-0.43 (-1.43, 0.55)	-0.02 (-0.4, 0.35)	0.29 (-0.06, 0.65)	-0.4 (-1.06, 0.27)
**IT**	0.2 (-0.11, 0.51)	-0.01 (-0.3, 0.28)	0.01 (0, 0.03)	-1.22 (-1.93, -0.53)	-0.31 (-0.67, 0.05)	0.25 (-0.11, 0.61)	0.1 (-0.77, 0.92)
**LT**	0.1 (-0.2, 0.39)	0.33 (0.05, 0.62)	0.01 (0, 0.02)	0.48 (-0.19, 1.15)	-0.12 (-0.45, 0.21)	0.01 (-0.35, 0.38)	-0.25 (-1.08, 0.59)
**LV**	-0.15 (-1.08, 0.77)	0.75 (-0.15, 1.7)	-0.01 (-0.05, 0.03)	-1.05 (-3.46, 1.33)	-0.08 (-1.16, 0.99)	0.55 (-0.47, 1.6)	-0.69 (-2.83, 1.39)
**NL**	-0.17 (-0.44, 0.1)	0.18 (-0.09, 0.46)	0.02 (0.01, 0.03)	-0.92 (-1.85, 0)	-0.69 (-1.02, -0.36)	-0.14 (-0.48, 0.21)	0.23 (-0.68, 1.13)
**PL**	-0.03 (-0.28, 0.21)	0.37 (0.13, 0.62)	0 (-0.01, 0.01)	-0.49 (-1.02, 0.04)	-0.56 (-0.89, -0.24)	0.04 (-0.28, 0.36)	-0.24 (-0.9, 0.42)
**PT**	0.19 (-0.07, 0.45)	0.18 (-0.07, 0.43)	0 (-0.01, 0.01)	0.04 (-0.65, 0.72)	0.11 (-0.19, 0.4)	0.47 (0.13, 0.8)	0.68 (0.01, 1.35)
**RO**	-0.15 (-0.48, 0.18)	0.42 (0.14, 0.7)	0.02 (0.01, 0.03)	0.1 (-0.65, 0.87)	-0.01 (-0.35, 0.34)	-0.07 (-0.45, 0.3)	-0.41 (-1.23, 0.4)
**SE**	-0.02 (-0.32, 0.28)	0.24 (-0.06, 0.53)	0.01 (0, 0.02)	-0.49 (-1.13, 0.15)	-0.37 (-0.74, -0.01)	0.11 (-0.29, 0.51)	0.13 (-0.53, 0.79)
**SI**	-0.29 (-0.64, 0.07)	0.36 (-0.01, 0.72)	0.01 (0, 0.02)	-0.84 (-1.79, 0.1)	-0.64 (-1.06, -0.22)	0.26 (-0.39, 0.92)	1.54 (0.25, 2.9)
**SK**	-0.37 (-0.64, -0.09)	0.16 (-0.12, 0.44)	0.02 (0.01, 0.03)	0.21 (-1.1, 1.55)	0.01 (-0.35, 0.38)	0.11 (-0.28, 0.51)	-0.2 (-1.47, 1.08)

Supplementary Table 9.

Coefficients of the linear model for every country for the statement "*Wolves that kill livestock should be killed*" (Q6.2), median values with 95% credible intervals (shown with a beige background when 0 is not included in the interval). All variables are categorical except age. Intercepts are rural, female and political center.

Countries	Rural – Urban	Female – Male	Age	Extreme left	Left	Right	Extreme right
**AT**	-0.25 (-0.49, -0.01)	0.01 (-0.23, 0.25)	0 (0, 0.01)	-0.44 (-1.82, 0.89)	-0.34 (-0.64, -0.04)	0.17 (-0.15, 0.49)	0.16 (-0.73, 1.05)
**BE**	0.12 (-0.13, 0.37)	0.17 (-0.08, 0.42)	0.01 (0, 0.02)	-0.42 (-1.1, 0.25)	-0.44 (-0.79, -0.1)	0.38 (0.08, 0.68)	-0.09 (-0.75, 0.56)
**BG**	-0.2 (-0.59, 0.19)	0.44 (0.17, 0.72)	0.02 (0.01, 0.03)	0.5 (-0.27, 1.28)	0.12 (-0.25, 0.5)	0.16 (-0.23, 0.53)	0.66 (-0.07, 1.39)
**CZ**	-0.02 (-0.24, 0.19)	0.19 (0, 0.38)	0.01 (0.01, 0.02)	0.01 (-0.67, 0.69)	0.41 (0.14, 0.7)	-0.06 (-0.31, 0.19)	0.04 (-0.67, 0.74)
**DE**	0 (-0.27, 0.27)	0.23 (-0.03, 0.49)	0.01 (0, 0.02)	-0.05 (-0.73, 0.62)	-0.2 (-0.53, 0.12)	0.05 (-0.32, 0.41)	0.42 (-0.98, 1.83)
**DK**	0.05 (-0.23, 0.34)	-0.01 (-0.29, 0.28)	0.01 (0, 0.02)	-1.33 (-1.83, -0.85)	0.01 (-0.34, 0.38)	0.1 (-0.29, 0.49)	1.55 (0.82, 2.31)
**EE**	-0.25 (-0.6, 0.09)	-0.14 (-0.49, 0.21)	0.03 (0.02, 0.05)	0.56 (-0.67, 1.8)	-0.16 (-0.63, 0.31)	0.31 (-0.09, 0.72)	1.44 (-0.22, 3.15)
**ES**	0.14 (-0.15, 0.44)	0.43 (0.13, 0.74)	0.02 (0.01, 0.03)	-0.58 (-1.2, 0.03)	-0.23 (-0.58, 0.11)	0.15 (-0.24, 0.54)	0.93 (-0.04, 1.9)
**FI**	-0.23 (-0.5, 0.03)	0.08 (-0.18, 0.34)	0.01 (0, 0.02)	-0.05 (-0.75, 0.65)	-0.42 (-0.76, -0.08)	0.25 (-0.09, 0.58)	-0.05 (-0.74, 0.67)
**FR**	-0.04 (-0.3, 0.21)	0.08 (-0.18, 0.33)	0.01 (0, 0.02)	-0.31 (-0.94, 0.32)	-0.25 (-0.57, 0.06)	-0.08 (-0.45, 0.28)	0.29 (-0.12, 0.69)
**GR**	-0.18 (-0.44, 0.09)	0.2 (-0.07, 0.47)	0.01 (0, 0.02)	-0.6 (-1.46, 0.23)	-0.35 (-0.71, 0.01)	0 (-0.33, 0.33)	0.54 (-0.08, 1.15)
**HR**	-0.14 (-0.52, 0.23)	0.21 (-0.12, 0.55)	0.01 (-0.01, 0.02)	-0.7 (-1.9, 0.48)	-0.1 (-0.49, 0.28)	0.46 (-0.01, 0.93)	0.13 (-0.85, 1.1)
**HU**	-0.07 (-0.33, 0.2)	0.09 (-0.19, 0.35)	0.01 (0, 0.02)	-0.25 (-1.19, 0.66)	-0.1 (-0.44, 0.24)	0.22 (-0.1, 0.55)	-0.22 (-0.84, 0.38)
**IT**	0.11 (-0.15, 0.37)	0.06 (-0.18, 0.3)	0.01 (0, 0.02)	-1.04 (-1.6, -0.49)	-0.41 (-0.71, -0.1)	0.16 (-0.15, 0.46)	0.57 (-0.13, 1.28)
**LT**	-0.06 (-0.35, 0.24)	0.22 (-0.06, 0.5)	0.02 (0.01, 0.03)	0.22 (-0.44, 0.88)	-0.01 (-0.33, 0.32)	-0.09 (-0.45, 0.27)	-0.68 (-1.52, 0.16)
**LV**	0.45 (-0.34, 1.24)	0.4 (-0.39, 1.21)	0.01 (-0.02, 0.05)	-0.6 (-2.77, 1.52)	0.53 (-0.42, 1.49)	0.28 (-0.6, 1.18)	-0.96 (-2.84, 0.87)
**NL**	-0.16 (-0.35, 0.02)	0.16 (-0.02, 0.34)	0.01 (0.01, 0.02)	-1.16 (-1.88, -0.5)	-0.49 (-0.73, -0.27)	0.1 (-0.13, 0.32)	-0.35 (-0.96, 0.25)
**PL**	0.07 (-0.17, 0.32)	0.13 (-0.11, 0.38)	0.01 (0, 0.02)	-0.53 (-1.07, 0)	-0.61 (-0.94, -0.28)	0.09 (-0.23, 0.42)	-0.09 (-0.75, 0.56)
**PT**	0.13 (-0.13, 0.39)	0.22 (-0.03, 0.47)	0.01 (0, 0.02)	-0.1 (-0.79, 0.57)	-0.05 (-0.34, 0.24)	0.23 (-0.1, 0.57)	0.17 (-0.5, 0.84)
**RO**	-0.11 (-0.42, 0.21)	0.31 (0.04, 0.58)	0.02 (0.01, 0.03)	0.33 (-0.42, 1.06)	0 (-0.34, 0.33)	-0.29 (-0.64, 0.08)	-0.53 (-1.31, 0.24)
**SE**	-0.11 (-0.4, 0.17)	0.32 (0.03, 0.61)	0.01 (0, 0.02)	-0.77 (-1.39, -0.15)	-0.32 (-0.68, 0.03)	0.31 (-0.07, 0.7)	0.31 (-0.32, 0.95)
**SI**	-0.24 (-0.61, 0.14)	0.1 (-0.29, 0.48)	0.02 (0.01, 0.03)	-0.83 (-1.82, 0.16)	-0.72 (-1.17, -0.28)	0.54 (-0.13, 1.23)	0.54 (-0.81, 1.88)
**SK**	-0.15 (-0.41, 0.12)	0.05 (-0.22, 0.32)	0.03 (0.02, 0.04)	0.78 (-0.46, 2.04)	-0.12 (-0.46, 0.23)	-0.07 (-0.44, 0.29)	-0.75 (-2, 0.47)

Supplementary Table 10.

Coefficients of the linear model for every country for the statement "*A lynx that kills livestock should be killed*" (Q6.3), median values with 95% credible intervals (shown with a beige background when 0 is not included in the interval). All variables are categorical except age. Intercepts are rural, female and political center.

Countries	Rural – Urban	Female – Male	Age	Extreme left	Left	Right	Extreme right
AT	-0.23 (-0.47, 0)	-0.05 (-0.29, 0.19)	0 (-0.01, 0.01)	-0.55 (-1.88, 0.75)	-0.32 (-0.62, -0.03)	0.1 (-0.22, 0.41)	-0.05 (-0.94, 0.82)
BE	0.11 (-0.14, 0.36)	0.27 (0.02, 0.52)	0.01 (0, 0.02)	-0.25 (-0.92, 0.42)	-0.47 (-0.8, -0.13)	0.39 (0.1, 0.69)	-0.15 (-0.79, 0.51)
BG	-0.21 (-0.59, 0.17)	0.39 (0.12, 0.65)	0.02 (0.01, 0.03)	0.63 (-0.12, 1.4)	0.13 (-0.24, 0.5)	0 (-0.37, 0.37)	0.6 (-0.11, 1.33)
CZ	0.02 (-0.21, 0.25)	0.07 (-0.13, 0.28)	0.01 (0, 0.02)	0.34 (-0.38, 1.09)	0.4 (0.12, 0.71)	-0.11 (-0.38, 0.16)	-0.08 (-0.86, 0.69)
DE	0.05 (-0.21, 0.31)	0.12 (-0.12, 0.37)	0 (-0.01, 0.01)	-0.22 (-0.88, 0.44)	-0.29 (-0.6, 0.02)	0.07 (-0.28, 0.4)	0.28 (-1.07, 1.61)
DK	0.04 (-0.25, 0.34)	-0.14 (-0.43, 0.15)	0.01 (0, 0.02)	-1.29 (-1.79, -0.79)	-0.01 (-0.38, 0.36)	0.1 (-0.3, 0.49)	1.72 (0.95, 2.53)
EE	-0.26 (-0.6, 0.07)	-0.07 (-0.41, 0.27)	0.03 (0.01, 0.04)	0.71 (-0.47, 1.9)	-0.13 (-0.6, 0.32)	0.26 (-0.12, 0.65)	1.56 (-0.05, 3.23)
ES	0.08 (-0.21, 0.36)	0.17 (-0.12, 0.47)	0.02 (0.01, 0.03)	-0.44 (-1.06, 0.16)	-0.17 (-0.51, 0.17)	0.31 (-0.07, 0.69)	0.78 (-0.13, 1.71)
FI	-0.19 (-0.46, 0.1)	-0.03 (-0.3, 0.25)	0 (-0.01, 0.01)	-0.04 (-0.78, 0.69)	-0.42 (-0.78, -0.07)	0.22 (-0.13, 0.57)	-0.17 (-0.9, 0.58)
FR	-0.11 (-0.37, 0.16)	0.01 (-0.26, 0.28)	0.01 (0, 0.02)	0.06 (-0.6, 0.73)	-0.15 (-0.48, 0.19)	-0.02 (-0.4, 0.36)	0.36 (-0.06, 0.79)
GR	-0.1 (-0.38, 0.17)	0.14 (-0.13, 0.42)	0.01 (0, 0.02)	-0.62 (-1.49, 0.24)	-0.32 (-0.69, 0.04)	0.01 (-0.33, 0.35)	0.49 (-0.14, 1.12)
HR	-0.21 (-0.59, 0.17)	0.27 (-0.06, 0.6)	0 (-0.01, 0.02)	-0.89 (-2.13, 0.32)	-0.25 (-0.63, 0.14)	0.54 (0.07, 1.01)	-0.14 (-1.12, 0.85)
HU	-0.09 (-0.36, 0.18)	0.01 (-0.26, 0.29)	0.01 (0, 0.02)	-0.31 (-1.26, 0.62)	-0.2 (-0.55, 0.15)	0.17 (-0.16, 0.51)	-0.31 (-0.95, 0.32)
IT	0.15 (-0.12, 0.43)	0.01 (-0.25, 0.26)	0.01 (0, 0.03)	-0.97 (-1.58, -0.38)	-0.37 (-0.69, -0.05)	0.23 (-0.09, 0.55)	0.38 (-0.37, 1.13)
LT	0.11 (-0.19, 0.41)	0.11 (-0.18, 0.4)	0.01 (0, 0.02)	0.21 (-0.46, 0.87)	-0.02 (-0.35, 0.31)	0.03 (-0.32, 0.39)	-0.68 (-1.53, 0.17)
LV	0.29 (-0.46, 1.05)	0.58 (-0.17, 1.34)	0.01 (-0.02, 0.05)	-0.45 (-2.51, 1.56)	0.74 (-0.14, 1.64)	0.11 (-0.74, 0.96)	-0.77 (-2.55, 0.96)
NL	-0.24 (-0.52, 0.04)	0.16 (-0.13, 0.44)	0.02 (0.01, 0.03)	-1.18 (-2.17, -0.2)	-0.69 (-1.03, -0.35)	0.2 (-0.16, 0.55)	-0.18 (-1.13, 0.75)
PL	0.06 (-0.2, 0.31)	0.22 (-0.03, 0.47)	0.01 (0, 0.02)	-0.68 (-1.24, -0.13)	-0.5 (-0.83, -0.17)	0.04 (-0.29, 0.37)	-0.43 (-1.1, 0.23)
PT	0.12 (-0.13, 0.38)	0.14 (-0.11, 0.39)	0.01 (0, 0.02)	-0.26 (-0.94, 0.42)	-0.1 (-0.39, 0.19)	0.21 (-0.12, 0.54)	-0.16 (-0.81, 0.49)
RO	-0.25 (-0.58, 0.07)	0.26 (-0.01, 0.54)	0.01 (0, 0.03)	0.52 (-0.23, 1.27)	0 (-0.34, 0.34)	-0.23 (-0.6, 0.13)	-0.05 (-0.83, 0.73)
SE	-0.12 (-0.4, 0.16)	0.18 (-0.1, 0.46)	0.01 (0, 0.02)	-0.78 (-1.38, -0.17)	-0.34 (-0.68, 0)	0.21 (-0.17, 0.58)	0.22 (-0.39, 0.84)
SI	-0.12 (-0.5, 0.26)	-0.15 (-0.54, 0.23)	0.02 (0, 0.03)	-1.06 (-2.12, -0.02)	-0.66 (-1.11, -0.22)	0.61 (-0.08, 1.3)	0.86 (-0.46, 2.21)
SK	-0.11 (-0.38, 0.16)	-0.08 (-0.36, 0.2)	0.02 (0.01, 0.03)	0.91 (-0.36, 2.22)	-0.07 (-0.43, 0.29)	-0.31 (-0.7, 0.08)	-0.67 (-1.94, 0.61)

Supplementary Table 11.

Coefficients of the linear model for every country for the statement "*How likely or unlikely you are to write to or call a politician to express your support for carnivore restoration efforts?*" (Q11), median values with 95% credible intervals (shown with a beige background when 0 is not included in the interval). All variables are categorical except age. Intercepts are rural, female and political center.

Countries	Rural – Urban	Female – Male	Age	Extreme left	Left	Right	Extreme right
AT	0.44 (0.14, 0.75)	0.49 (0.19, 0.8)	0 (-0.02, 0.01)	1.27 (-0.36, 2.88)	0.17 (-0.2, 0.53)	-0.08 (-0.49, 0.33)	0.21 (-0.86, 1.28)
BE	0.26 (-0.02, 0.54)	0.03 (-0.25, 0.3)	0 (-0.01, 0.01)	0.49 (-0.22, 1.21)	-0.23 (-0.61, 0.14)	-0.31 (-0.65, 0.03)	-0.13 (-0.87, 0.58)
BG	-0.23 (-0.51, 0.04)	-0.15 (-0.35, 0.05)	0 (-0.01, 0.01)	0.33 (-0.21, 0.89)	0.13 (-0.14, 0.4)	-0.12 (-0.39, 0.15)	-0.25 (-0.79, 0.27)
CZ	0.09 (-0.22, 0.41)	0.24 (-0.03, 0.54)	-0.01 (-0.02, 0)	0.08 (-0.94, 1.09)	-0.45 (-0.9, -0.04)	-0.38 (-0.78, -0.01)	0.23 (-0.76, 1.21)
DE	0.49 (0.19, 0.8)	-0.1 (-0.39, 0.19)	-0.02 (-0.03, -0.01)	0.86 (0.13, 1.59)	-0.07 (-0.44, 0.28)	-0.07 (-0.48, 0.33)	1.44 (-0.1, 3.03)
DK	0.08 (-0.2, 0.35)	0.1 (-0.17, 0.38)	-0.02 (-0.03, -0.01)	-0.14 (-0.59, 0.3)	-0.16 (-0.5, 0.18)	-0.34 (-0.72, 0.03)	-0.45 (-1.13, 0.22)
EE	-0.25 (-0.65, 0.14)	0.25 (-0.14, 0.65)	0 (-0.01, 0.02)	0.53 (-0.85, 1.91)	-0.43 (-0.99, 0.12)	0.02 (-0.43, 0.47)	0.2 (-1.65, 2.03)
ES	0.18 (-0.09, 0.45)	0.24 (-0.04, 0.52)	0 (-0.01, 0.01)	0.18 (-0.38, 0.74)	0.05 (-0.28, 0.37)	-0.13 (-0.51, 0.24)	-0.13 (-1.05, 0.78)
FI	0.18 (-0.16, 0.51)	0.2 (-0.12, 0.52)	0 (-0.01, 0.01)	-0.47 (-1.4, 0.43)	0.05 (-0.37, 0.47)	-0.37 (-0.8, 0.04)	0.1 (-0.76, 0.95)
FR	-0.06 (-0.33, 0.21)	0.23 (-0.03, 0.51)	-0.01 (-0.02, 0)	0.23 (-0.44, 0.89)	0.17 (-0.17, 0.5)	0.03 (-0.36, 0.41)	-0.23 (-0.67, 0.2)
GR	-0.06 (-0.32, 0.21)	0.04 (-0.22, 0.31)	0.01 (-0.01, 0.02)	0.08 (-0.73, 0.9)	0.26 (-0.09, 0.61)	-0.04 (-0.37, 0.29)	-0.36 (-1, 0.28)
HR	0.13 (-0.26, 0.51)	0.03 (-0.29, 0.36)	0 (-0.01, 0.02)	-0.2 (-1.35, 0.91)	-0.2 (-0.58, 0.17)	-0.87 (-1.38, -0.38)	-0.25 (-1.22, 0.68)
HU	-0.06 (-0.35, 0.23)	0.16 (-0.13, 0.46)	0 (-0.01, 0.01)	0.23 (-0.74, 1.19)	0.11 (-0.26, 0.49)	0.04 (-0.32, 0.4)	0.1 (-0.59, 0.78)
IT	0.48 (0.22, 0.75)	0.18 (-0.07, 0.43)	0 (-0.01, 0.01)	0.55 (0.03, 1.07)	0.12 (-0.19, 0.42)	0.03 (-0.28, 0.35)	-0.44 (-1.19, 0.3)
LT	-0.26 (-0.57, 0.06)	0.14 (-0.16, 0.45)	-0.02 (-0.03, -0.01)	-0.6 (-1.35, 0.12)	0 (-0.36, 0.35)	-0.09 (-0.48, 0.3)	0.15 (-0.79, 1.06)
LV	0.1 (-0.54, 0.75)	-0.02 (-0.66, 0.62)	-0.01 (-0.03, 0.02)	0.52 (-1.13, 2.18)	-0.25 (-1.01, 0.48)	-0.31 (-1.06, 0.38)	1.06 (-0.29, 2.49)
NL	0.05 (-0.23, 0.35)	0.34 (0.04, 0.63)	-0.01 (-0.02, 0)	0.9 (-0.01, 1.8)	-0.12 (-0.46, 0.23)	-0.65 (-1.03, -0.28)	-0.84 (-1.84, 0.13)
PL	0.37 (0.11, 0.62)	-0.1 (-0.35, 0.15)	0 (-0.01, 0.01)	0.44 (-0.09, 0.98)	0.2 (-0.13, 0.53)	0.25 (-0.08, 0.58)	0.46 (-0.21, 1.13)
PT	-0.1 (-0.38, 0.18)	-0.03 (-0.3, 0.24)	0 (-0.01, 0.01)	-0.01 (-0.73, 0.7)	0.02 (-0.29, 0.34)	-0.23 (-0.59, 0.12)	0.26 (-0.45, 0.96)
RO	-0.02 (-0.25, 0.22)	0.11 (-0.09, 0.31)	0 (-0.01, 0.01)	0.54 (0.02, 1.08)	-0.02 (-0.26, 0.22)	0.13 (-0.13, 0.4)	0.3 (-0.24, 0.85)
SE	0.53 (0.25, 0.81)	0.03 (-0.25, 0.3)	0 (-0.01, 0.01)	0.38 (-0.21, 0.97)	0.15 (-0.18, 0.49)	-0.21 (-0.58, 0.16)	-0.02 (-0.63, 0.58)
SI	0.23 (-0.13, 0.6)	0.12 (-0.25, 0.49)	0.01 (0, 0.02)	0.66 (-0.26, 1.58)	0.06 (-0.37, 0.48)	-0.03 (-0.69, 0.61)	0.88 (-0.28, 2.08)
SK	-0.03 (-0.34, 0.28)	0.35 (0.04, 0.67)	0 (-0.02, 0.01)	0.28 (-1.11, 1.63)	0.07 (-0.33, 0.48)	0.09 (-0.34, 0.52)	0.06 (-1.32, 1.4)

Supplementary Table 12.

Coefficients of the linear model for every country for the statement "*How likely or unlikely you are to write to or call a politician to express your opposition for carnivore restoration efforts?*" (Q12), median values with 95% credible intervals (shown with a beige background when 0 is not included in the interval). All variables are categorical except age. Intercepts are rural, female and political center.

Countries	Rural – Urban	Female – Male	Age	Extreme left	Left	Right	Extreme right
**AT**	0.13 (-0.26, 0.53)	0.47 (0.08, 0.88)	-0.01 (-0.03, 0)	1.02 (-0.88, 2.88)	-0.24 (-0.73, 0.25)	0.2 (-0.31, 0.71)	0.32 (-1.02, 1.65)
**BE**	0.32 (0.01, 0.64)	0.39 (0.08, 0.72)	-0.02 (-0.03, -0.01)	-1.32 (-2.37, -0.35)	-0.51 (-0.95, -0.08)	-0.2 (-0.58, 0.18)	0.61 (-0.17, 1.4)
**BG**	-0.17 (-0.49, 0.14)	-0.04 (-0.27, 0.18)	0.01 (0, 0.02)	0.33 (-0.28, 0.96)	-0.04 (-0.36, 0.27)	-0.09 (-0.41, 0.21)	-0.59 (-1.29, 0.05)
**CZ**	-0.09 (-0.46, 0.28)	0.26 (-0.06, 0.61)	0 (-0.01, 0.01)	0.6 (-0.54, 1.78)	-0.05 (-0.53, 0.43)	-0.26 (-0.72, 0.17)	0.63 (-0.51, 1.79)
**DE**	0.24 (-0.1, 0.57)	0.28 (-0.03, 0.6)	-0.02 (-0.03, -0.01)	0.77 (-0.03, 1.58)	-0.56 (-0.96, -0.15)	-0.11 (-0.55, 0.32)	0.68 (-0.93, 2.31)
**DK**	-0.04 (-0.34, 0.26)	0.14 (-0.16, 0.44)	-0.02 (-0.03, -0.01)	-1.29 (-1.86, -0.74)	-0.26 (-0.64, 0.11)	-0.22 (-0.64, 0.19)	0.5 (-0.19, 1.19)
**EE**	-0.03 (-0.45, 0.39)	0.31 (-0.11, 0.74)	0 (-0.02, 0.01)	0.86 (-0.57, 2.27)	-0.08 (-0.66, 0.49)	-0.24 (-0.74, 0.25)	-0.33 (-2.28, 1.57)
**ES**	0.13 (-0.17, 0.43)	0.47 (0.16, 0.78)	-0.01 (-0.02, 0)	-0.4 (-1.05, 0.23)	-0.19 (-0.56, 0.17)	-0.15 (-0.57, 0.26)	0.24 (-0.74, 1.2)
**FI**	0.01 (-0.45, 0.46)	0.26 (-0.17, 0.71)	-0.01 (-0.03, 0.01)	-1.28 (-2.76, 0.06)	-0.56 (-1.16, 0.01)	-0.18 (-0.73, 0.36)	0.35 (-0.8, 1.47)
**FR**	0.1 (-0.18, 0.38)	0.36 (0.07, 0.64)	-0.01 (-0.02, 0)	0.06 (-0.64, 0.75)	-0.09 (-0.45, 0.26)	-0.01 (-0.41, 0.39)	-0.23 (-0.68, 0.23)
**GR**	0.18 (-0.1, 0.46)	0.06 (-0.23, 0.33)	-0.01 (-0.02, 0.01)	-0.93 (-1.87, -0.04)	0 (-0.38, 0.37)	-0.16 (-0.51, 0.19)	-0.19 (-0.86, 0.47)
**HR**	-0.09 (-0.57, 0.38)	0.31 (-0.1, 0.73)	-0.01 (-0.03, 0.01)	-2.18 (-4.37, -0.36)	-0.27 (-0.75, 0.2)	-0.33 (-0.94, 0.26)	0.07 (-1.1, 1.22)
**HU**	-0.03 (-0.37, 0.31)	0.15 (-0.2, 0.5)	0 (-0.01, 0.01)	0.72 (-0.36, 1.83)	0.06 (-0.39, 0.5)	0.06 (-0.37, 0.49)	0.79 (0.03, 1.55)
**IT**	0.59 (0.26, 0.92)	0.15 (-0.15, 0.46)	0 (-0.01, 0.02)	0.18 (-0.47, 0.82)	-0.12 (-0.5, 0.25)	0.11 (-0.27, 0.49)	0.3 (-0.57, 1.15)
**LT**	-0.01 (-0.38, 0.35)	0.55 (0.2, 0.91)	-0.01 (-0.02, 0.01)	-0.42 (-1.28, 0.42)	-0.01 (-0.42, 0.4)	-0.26 (-0.71, 0.2)	-0.31 (-1.41, 0.76)
**LV**	-0.26 (-0.98, 0.42)	0.12 (-0.59, 0.83)	-0.01 (-0.05, 0.01)	0.17 (-1.59, 1.92)	-0.03 (-0.85, 0.81)	-0.4 (-1.25, 0.38)	1.09 (-0.41, 2.64)
**NL**	0.05 (-0.29, 0.39)	0.22 (-0.12, 0.57)	-0.01 (-0.02, 0)	0.18 (-0.94, 1.26)	-0.38 (-0.79, 0.04)	-0.3 (-0.73, 0.13)	0.43 (-0.64, 1.49)
**PL**	0.33 (0.05, 0.62)	-0.1 (-0.38, 0.18)	0 (-0.01, 0.01)	0.07 (-0.54, 0.67)	0 (-0.37, 0.37)	0.45 (0.09, 0.81)	-0.08 (-0.87, 0.68)
**PT**	-0.01 (-0.35, 0.33)	0.22 (-0.11, 0.54)	0.01 (0, 0.02)	0.62 (-0.25, 1.49)	0.02 (-0.37, 0.4)	0.04 (-0.39, 0.48)	0.2 (-0.67, 1.04)
**RO**	-0.09 (-0.36, 0.17)	0.3 (0.07, 0.54)	-0.01 (-0.02, 0)	0.64 (0.07, 1.26)	-0.02 (-0.29, 0.26)	-0.11 (-0.42, 0.19)	0.01 (-0.62, 0.64)
**SE**	0.35 (0.05, 0.65)	0.24 (-0.06, 0.55)	-0.01 (-0.02, 0)	-0.02 (-0.68, 0.64)	0 (-0.37, 0.37)	-0.17 (-0.57, 0.22)	0.3 (-0.35, 0.95)
**SI**	-0.06 (-0.49, 0.38)	0.33 (-0.1, 0.78)	0.02 (0.01, 0.04)	-0.29 (-1.5, 0.83)	-0.11 (-0.63, 0.4)	0.56 (-0.17, 1.3)	1.14 (-0.19, 2.49)
**SK**	-0.27 (-0.65, 0.09)	0.35 (-0.03, 0.73)	0 (-0.01, 0.01)	0.45 (-1.15, 2.05)	0.03 (-0.45, 0.5)	-0.23 (-0.78, 0.3)	-1.23 (-3.4, 0.7)

Supplementary Survey Translations

Survey questions in Bulgarian

Q1: През последните десетилетия в някои части на Европа отново се заселват големи хищници, като вълци, кафяви мечки и рисове. Като цяло подкрепяте ли или сте против възстановяването на популациите от едри хищници? (*Изцяло против / Против / Неутрално / Не знам със сигурност / Подкрепям ги / Изцяло подкрепям*)

Q2: Според мен популациите на вълците в Европа трябва да бъдат... (*Намалени значително / Намалени / Да останат както са / Увеличени / Увеличени значително / Не знам*)

Q3: Според мен популациите на рисовете в Европа трябва да бъдат... (*Намалени значително / Намалени / Да останат както са / Увеличени / Увеличени значително / Не знам*)

Q4: Според мен популациите на кафявите мечки в Европа трябва да бъдат... (*Намалени значително / Намалени / Да останат както са / Увеличени / Увеличени значително / Не знам*)

Q5: Като цяло подкрепяте ли или сте против лова на едри хищници? (*Изцяло против / Против / Неутрално/Не знам със сигурност / Подкрепям ги / Изцяло подкрепям*)

Q6: Доколко сте съгласни или несъгласни със следното:

1. Ако мечка нападне човек, тя трябва да бъде убита независимо от обстоятелствата.
2. Вълците, които убиват добитък, трябва да бъдат убивани.
3. Рис, който убива добитък, трябва да бъде убит.

(*Напълно съгласни / Донякъде съгласни / Нито съгласни, нито несъгласни / Донякъде несъгласни / Напълно несъгласни*)

Q7: Какъв е полът Ви? (*Мъж / Жена*)

Q8: Въведете годината си на раждане (във формат ГГГГ)

Q9: Как бихте описали настоящото си местожителство или общност? (*Голям урбанизиран район / Град / Село / Отдалечена къща*)

Q10: Когато става дума за политика, моля, посочете към кое от посочените по-долу се причислявате. (*Крайно ляво / Ляво (социалдемократическо) / Център / Дясно (консервативно) / Крайно дясно*)

Q11: По-долу са изброени редица действия, които можете да предприемете, за да подкрепите опазването на едрите хищници в държавата Ви. Моля, посочете доколко е вероятно или не е… Да пишете или да се обадите на политик, за да изразите подкрепата си за усилията за възстановяване на популация на хищници. (*Много малко вероятно / Малко вероятно / Не мога да определя / Донякъде вероятно / Много е вероятно*)

Q12: По-долу са изброени редица действия, които бихте могли да предприемете, за да се противопоставите на големи популации от хищници в държавата си. Моля, посочете доколко е вероятно или не е... Да пишете или да се обадите на политик, за да изразите несъгласието си с усилията за възстановяване на популации на хищници. (*Много малко вероятно / Малко вероятно / Не мога да определя / Донякъде вероятно / Много е вероятно*)

Survey questions in Croatian

Q1: Veliki mesojedi poput vuka, smeđeg medvjeda i risa posljednjih desetljeća ponovo naseljavaju dijelove Europe. Općenito govoreći, biste li rekli da podržavate ili se protivite oporavku vrsta velikih mesojeda? (*Izrazito se protivim / Protivim se / Nemam određeno mišljenje ili nisam siguran / Podržavam / Izrazito podržavam*)

Q2: Po mojem mišljenju, populaciju vukova u Europi treba... (*Uvelike smanjiti / Smanjiti / Ostaviti kakva jest / Povećati / Uvelike povećati / Ne znam*)

Q3: Po mojemu mišljenju, populaciju risova u Europi treba... (*Uvelike smanjiti / Smanjiti / Ostaviti kakva jest / Povećati / Uvelike povećati / Ne znam*)

Q4: Po mojemu mišljenju, populaciju smeđih medvjeda u Europi treba... (*Uvelike smanjiti / Smanjiti / Ostaviti kakva jest / Povećati / Uvelike povećati / Ne znam*)

Q5: Općenito govoreći, biste li rekli da podržavate ili se protivite lovu na velike mesojede? (*Izrazito se protivim / Protivim se / Nemam određeno mišljenje ili nisam siguran / Podržavam / Izrazito podržavam*)

Q6: U kojoj se mjeri slažete ili ne slažete sa sljedećim::

1. Ako medvjed napadne čovjeka, tog medvjeda treba ubiti neovisno o okolnostima.
2. Vukove koji ubijaju stoku treba ubiti.
3. Risove koji ubijaju stoku treba ubiti.

(*Izrazito se slažem / Donekle se slažem / Niti se slažem niti se ne slažem / Donekle se ne slažem / Izrazito se ne slažem*)

Q7: Kojeg ste spola? (*Muško / Žensko*)

Q8: Ovdje upišite godinu rođenja (u formatu GGGG)

Q9: Kako biste opisali svoje trenutačno mjesto prebivališta ili zajednicu? (*Veliko urbano područje / Grad / Selo / Kuća na osami*)

Q10: Kada je riječ o politici, naznačite što od sljedećeg vrijedi za vas. (*Krajnji ljevičar / Ljevičar (socijaldemokrat) / Centar / Desničar (konzervativac) / Krajnji desničar*)

Q11: U nastavku je određeni broj aktivnosti koje možete poduzeti da biste podržali očuvanje velikih mesojeda u svojoj zemlji. Naznačite koliko je ili nije vjerojatno da ćete (se)... Pisati ili obratiti se političaru da biste iskazali podršku naporima za oporavak vrsta mesojeda. (*Nimalo vjerojatno / Donekle je nevjerojatno / Nisam siguran / Donekle je vjerojatno / Vrlo vjerojatno*)

Q12: U nastavku je određeni broj aktivnosti koje možete poduzeti da biste se usprotivili očuvanju velikih mesojeda u svojoj zemlji. Naznačite koliko je ili nije vjerojatno da ćete (se)... Pisati ili obratiti se političaru da biste iskazali protivljenje naporima za oporavak vrsta mesojeda. (*Nimalo vjerojatno / Donekle je nevjerojatno / Nisam siguran / Donekle je vjerojatno / Vrlo vjerojatno*)

Survey questions in Czech

Q1: Velké šelmy, jako jsou vlci, medvědi hnědí a rysi, v posledních desetiletích znovu osidlují některé části Evropy. Obecně byste řekli, že podporujete opětovný nárůst populace velkých šelem nebo mu oponujete? (*Rozhodně oponuji / Oponuji / Neutrální/nevím / Podporuji / Rozhodně podporuji*)

Q2: Dle mého názoru by populace vlků v Evropě měla být... (*Výrazně snížena / Snížena / Zůstat zhruba stejná / Zvýšena / Výrazně zvýšena / Nevím*)

Q3: Dle mého názoru by populace rysů v Evropě měla být... (*Výrazně snížena / Snížena / Zůstat zhruba stejná / Zvýšena / Výrazně zvýšena / Nevím*)

Q4: Dle mého názoru by populace medvědů hnědých v Evropě měla být... (*Výrazně snížena / Snížena / Zůstat zhruba stejná / Zvýšena / Výrazně zvýšena / Nevím*)

Q5: Obecně byste řekli, že podporujete lov velkých šelem nebo mu oponujete? (*Rozhodně oponuji / Oponuji / Neutrální/nevím / Podporuji / Rozhodně podporuji*)

Q6: Do jaké míry souhlasíte či nesouhlasíte s níže uvedenými výroky:

1. Pokud medvěd napadne člověka, měl by být usmrcen bez ohledu na okolnosti.
2. Vlci, kteří zabíjejí hospodářská zvířata, by měli být usmrceni.
3. Rys, který zabije hospodářská zvířata, by měl být usmrcen.

(*Rozhodně souhlasím / Spíše souhlasím / Ani souhlasím, ani nesouhlasím / Spíše nesouhlasím / Rozhodně nesouhlasím*)

Q7: Jaké je Vaše pohlaví? (*Muž / Žena*)

Q8: Zadejte rok, ve kterém jste se narodili (ve formátu RRRR)

Q9: Jak byste popsali své současné bydliště nebo oblast, ve které žijete? (*Velká městská oblast / Město / Vesnice / Dům na samotě*)

Q10: Pokud jde o politiku, uveďte, kam byste sami sebe zařadili. (*Extrémní levice / Levice (sociální demokrat) / Střed / Pravice (konzervativní) / Extrémní pravice*)

Q11: Níže je uvedena řada opatření, kterými můžete podpořit ochranu velkých šelem ve Vaší zemi. Uveďte, jak pravděpodobné nebo nepravděpodobné je, že... Napíšete nebo zavoláte politikovi a vyjádříte svou podporu úsilí o obnovu populace šelem. (*Velmi nepravděpodobné / Spíše nepravděpodobné / Nejsem rozhodnutý/á / Spíše pravděpodobné / Velmi pravděpodobné*)

Q12: Níže je uvedena řada opatření, kterými můžete oponovat rozšíření populací šelem ve Vaší zemi. Uveďte, jak pravděpodobné nebo nepravděpodobné je, že... Napíšete nebo zavoláte politikovi a vyjádříte svůj odpor vůči úsilí o obnovu populace šelem. (*Velmi nepravděpodobné / Spíše nepravděpodobné / Nejsem rozhodnutý/á / Spíše pravděpodobné / Velmi pravděpodobné*)

Survey questions in Danish

Q1: Store kødædende dyr som ulve, brune bjørne og losser har rekoloniseret dele af Europa i de seneste årtier. Generelt vil du sige, at du støtter eller er imod bedring af forholdene for store kødædende dyr? (*Stærkt imod / Imod / Neutral eller ikke sikker / Støtter / Støtter fuldt ud*)

Q2: Efter min mening bør ulvebestande I Europa … (*Reduceres kraftigt / Reduceres / Forblive nogenlunde uændret / Forøges / Forøges kraftigt / Det ved jeg ikke*)

Q3: Efter min mening bør bestanden af losser I Europa … (*Reduceres kraftigt / Reduceres / Forblive nogenlunde uændret / Forøges / Forøges kraftigt / Det ved jeg ikke*)

Q4: Efter min mening bør bestande af brune bjørne I Europa … (Reduceres kraftigt / Reduceres / Forblive nogenlunde uændret / Forøges / Forøges kraftigt / Det ved jeg ikke)

Q5: Generelt vil du sige, at du støtter eller er imod jagt på store kødædende dyr? (*Stærkt imod / Imod / Neutral eller ikke sikker / Støtter / Støtter fuldt ud*)

Q6: I hvilken grad er du enig i det følgende:
1. Hvis en bjørn angriber en person, skal den bjørn aflives uanset omstændighederne.
2. Ulve, der dræber husdyr, bør aflives.
3. En los, der dræber husdyr, bør aflives.

(*Meget enig / I nogen grad enig / Hverken enig eller uenig / I nogen grad uenig / Meget uenig*)

Q7: Hvad er dit køn? (*Mand / Kvinde*)

Q8: Indtast det år du blev født (i ÅÅÅÅ format)

Q9: Hvordan vil du beskrive din nuværende bolig eller lokalsamfund? (*Stort byområde / By / Landsby / Isoleret hus*)

Q10: Når det kommer til politik, bedes du angive, hvordan du ser dig selv. (*Ekstrem venstre / Venstre (socialdemokrat) / Centrum / Højre (konservativ) / Ekstrem højre*)

Q11: Nedenfor er en række handlinger, du kan udføre for at støtte bevarelsen af store kødædende dyr i dit land. Angiv, hvor sandsynligt eller usandsynligt, det er at du vil … Skrive til eller ringe til en politiker for at udtrykke din støtte til bestræbelserne på at genoprette forholdene for kødædende dyr. (*Meget usandsynligt / Usandsynligt i nogen grad / Har ikke besluttet mig / Sandsynligt i nogen grad / Meget sandsynligt*)

Q12: Nedenfor er en række handlinger, du kan udføre for at modsætte dig bevarelsen af bestanden af store kødædende dyr i dit land. Angiv, hvor sandsynligt eller usandsynligt, det er at du vil … Skrive til eller ringe til en politiker for at udtrykke din modstand mod bestræbelser på at genoprette forholdene for kødædende dyr. (*Meget usandsynligt / Usandsynligt i nogen grad / Har ikke besluttet mig / Sandsynligt i nogen grad / Meget sandsynligt*)

Survey questions in Dutch

Q1: Grote carnivoren zoals wolven, bruine beren en lynxen hebben zich de afgelopen decennia opnieuw gevestigd in Europa. Zou je in het algemeen zeggen dat je voor of tegen het herstel van grote carnivoren bent? (*Sterk tegen / Tegen / Neutraal of niet zeker / Voor / Sterk voor*)

Q2: Naar mijn mening moeten wolvenpopulaties in Europa … (*Sterk worden ingeperkt / Worden ingeperkt / Ongeveer gelijk blijven / Toenemen / Sterk toenemen / Weet ik niet*)

Q3: Naar mijn mening moeten lynxpopulaties in Europa ... (*Sterk worden ingeperkt / Worden ingeperkt / Ongeveer gelijk blijven / Toenemen / Sterk toenemen / Weet ik niet*)

Q4: Naar mijn mening moeten populaties bruine beren in Europa … (*Sterk worden ingeperkt / Worden ingeperkt / Ongeveer gelijk blijven / Toenemen / Sterk toenemen / Weet ik niet*)

Q5: Zou je in het algemeen zeggen dat je voor of tegen de jacht op grote carnivoren bent? (*Sterk tegen / Tegen / Neutraal of niet zeker / Voor / Sterk voor*)

Q6: In hoeverre ben je het eens of oneens met de volgende stellingen:

1. Als een beer een persoon aanvalt, moet die beer ongeacht de omstandigheden worden gedood.
2. Wolven die vee doden, moeten worden gedood.
3. Een lynx die vee doodt, moet worden gedood.

(*Zeer mee eens / Enigszins mee eens / Noch mee eens, noch mee oneens / Enigszins mee oneens / Zeer mee oneens*)

Q7: Wat is je geslacht/genderidentiteit? (*Man / Vrouw*)

Q8: Vul het jaartal in van het jaar waarin je bent geboren (in de indeling JJJJ)

Q9: Hoe zou je je huidige woonplaats of gemeenschap omschrijven? (*Groot stedelijk gebied / Stad / Dorp / Afgelegen huis*)

Q10: Geef aan hoe jij jezelf ziet als het om politiek gaat. (*Extreem links / Links (sociaaldemocraat) / Midden / Rechts (conservatief) / Extreem rechts*)

Q11: Hieronder vind je een aantal acties die je kunt ondernemen om het behoud van grote carnivoren in jouw land te steunen. Geef aan hoe waarschijnlijk of onwaarschijnlijk het is dat je ... Een politicus schrijft of belt om je steun te betuigen aan de inspanningen voor het herstel van carnivoren. (*Zeer onwaarschijnlijk / Enigszins onwaarschijnlijk / Weet ik nog niet / Enigszins waarschijnlijk / Zeer waarschijnlijk*)

Q12: Hieronder vind je een aantal acties die je kunt ondernemen om te laten zien dat je tegen grote carnivoorpopulaties in jouw land bent. Geef aan hoe waarschijnlijk of onwaarschijnlijk het is dat je ... Een politicus schrijft of belt om aan te geven dat je tegen de inspanningen voor het herstel van carnivoren bent. (*Zeer onwaarschijnlijk / Enigszins onwaarschijnlijk / Weet ik nog niet / Enigszins waarschijnlijk / Zeer waarschijnlijk*)

Survey questions in Estonian

Q1: Suurkiskjad nagu hundid, pruunkarud ja ilvesed on viimastel aastakümnetel asustanud taas osa Euroopast. Kas toetate üldiselt suurkiskjate asurkonna taastumist või olete selle vastu? (*Olen tugevalt vastu / Olen vastu / Erapooletu või pole kindel / Toetan / Toetan kindlalt*)

Q2: Minu arvates peaksid hundi asurkonnad Euroopas … (*olema suurel määral vähendatud / olema vähendatud / jääma umbes samale arvukusele / olema suurendatud / olema suurel määral suurendatud / Ma ei tea*)

Q3: Minu arvates peaksid ilveste asurkonnad Euroopas … (*olema suurel määral vähendatud / olema vähendatud / jääma umbes samale arvukusele / olema suurendatud / olema suurel määral suurendatud / Ma ei tea*)

Q4: Minu arvates peaksid pruunkaru asurkonnad Euroopas … (*olema suurel määral vähendatud / olema vähendatud / jääma umbes samale arvukusele / olema suurendatud / olema suurel määral suurendatud / Ma ei tea*)

Q5: Kas te üldiselt toetate suurkiskjate küttimist või olete selle vastu? (*Olen tugevalt vastu / Olen vastu / Erapooletu või pole kindel / Toetan / Toetan kindlalt*)

Q6: Millisel määral nõustute või ei nõustu järgmisega:
1. Kui karu inimest ründab, tuleks see karu asjaoludest hoolimata tappa.
2. Hundid, kes tapavad kariloomi, tuleks tappa.
3. Ilves, kes tapab kariloomi, tuleks tappa.

(*Nõustun kindlalt / Nõustun osaliselt / Jään neutraalsele seisukohale / Ei nõustu osaliselt / Ei nõustu üldse*)

Q7: Mis on teie sugu? (*Mees / Naine*)

Q8: Sisestage oma sünniaasta (AAAA vormingus)

Q9: Kuidas kirjeldaksite oma praegust elukohta või kogukonda? (*Suur linnapiirkond / Linn / Alevik / Maja eraldatud kohas*)

Q10: Mõeldes poliitikale; palun märkige, milliste alljärgnevate vaadetega ennast seostate. (*Äärmuslik vasakpoolne / Vasakpoolne (sotsiaaldemokraat) / Keskmik / Parempoolne (konservatiivne) / Paremäärmuslane*)

Q11: Allpool on esitatud mitmed tegevused, mida saate teha, et toetada suurkiskjate säilimist teie riigis. Palun märkige, kui tõenäoliselt või ebatõenäoliselt te... kirjutaksite või helistaksite poliitikule, et väljendada oma toetust kiskjate ennistamiseks tehtavatele jõupingutustele. (*Väga ebatõenäoline / Mõnevõrra ebatõenäoline / Pole kindel / Mõnevõrra tõenäoline / Väga tõenäoline*)

Q12: Allpool on toodud mitu toimingut, mida saate oma riigis teha selleks, et olla vastu suurkiskjate asurkondadele. Palun märkige, kui tõenäoliselt või ebatõenäoliselt te... kirjutaksite või helistaksite poliitikule, et väljendada oma vastuseisu kiskjate ennistamiseks tehtavaile jõupingutustele. (*Väga ebatõenäoline / Mõnevõrra ebatõenäoline / Pole kindel / Mõnevõrra tõenäoline / Väga tõenäoline*)

Survey questions in Finnish

Q1: Suurpedot, kuten sudet, ruskeakarhut ja ilvekset, ovat levittäytyneet uudelleen Euroopan osiin viime vuosikymmeninä. Yleisesti ottaen sanoisitko kannattavasi vai vastustavasi suurpetokantojen elpymistä? (*Vastustan jyrkästi / Vastustan / Ei kantaa tai en osaa sanoa / Kannatan / Kannatan vahvasti*)

Q2: Mielestäni susipopulaatioiden Euroopassa pitäisi… (*Vähentyä suuresti / Vähentyä / Pysyä suunnilleen samana / Lisääntyä / Lisääntyä suuresti / En tiedä*)

Q3: Mielestäni ilvespopulaatioiden Euroopassa pitäisi…(*Vähentyä suuresti / Vähentyä / Pysyä suunnilleen samana / Lisääntyä / Lisääntyä suuresti / En tiedä*)

Q4: Mielestäni ruskeakarhupopulaatioiden Euroopassa pitäisi… (*Vähentyä suuresti / Vähentyä / Pysyä suunnilleen samana / Lisääntyä / Lisääntyä suuresti / En tiedä*)

Q5: Yleisesti ottaen sanoisitko kannattavasi vai vastustavasi suurpetojen metsästystä? (*Vastustan jyrkästi / Vastustan / Ei kantaa tai en osaa sanoa / Kannatan / Kannatan vahvasti*)

Q6: Missä määrin olet samaa tai eri mieltä seuraavista:

1. Jos karhu hyökkää ihmisen kimppuun, karhu tulee tappaa olosuhteista riippumatta.
2. Sudet, jotka tappavat karjaa, on lopetettava.
3. Ilves, joka tappaa karjaa, on lopetettava.

(*Vahvasti samaa mieltä* / *Jokseenkin samaa mieltä / En samaa enkä eri mieltä / Jokseenkin eri mieltä / Vahvasti eri mieltä*)

Q7: Mikä on sukupuolesi? (*Mies / Nainen*)

Q8: Syötä syntymävuotesi (vuosi muodossa VVVV)

Q9: Miten kuvailisit nykyistä asuinpaikkaasi tai -yhteisöäsi? (*Suurkaupunkialue / Kaupunki / Kylä / Syrjäinen maaseutu*)

Q10: Mitä tulee politiikkaan, kerro, mihin seuraavista ajattelet kuuluvasi. (*Äärivasemmisto / Vasemmisto (sosialidemokraatti) / Keskusta / Oikeisto (konservatiivi) / Äärioikeisto*)

Q11: Alla on esitetty useita toimia, joita voit tehdä tukeaksesi suurpetojen suojelua maassasi. Kerro, kuinka todennäköisesti tai epätodennäköisesti... Kirjoitat tai soitat poliitikolle ilmaistaksesi tukesi petoeläinkantojen ennallistamispyrkimyksille. (*Erittäin epätodennäköisesti / Jokseenkin epätodennäköisesti / En osaa sanoa / Jokseenkin todennäköisesti / Erittäin todennäköisesti*)

Q12: Alla on esitetty useita toimia, joita voit tehdä vastustaaksesi suurpetopopulaatioita maassasi. Ilmoita kuinka todennäköisesti tai epätodennäköisesti... Kirjoitat tai soitat poliitikolle ilmaistaksesi vastustavasi petoeläinkantojen ennallistamispyrkimyksiä. (*Erittäin epätodennäköisesti / Jokseenkin epätodennäköisesti / En osaa sanoa / Jokseenkin todennäköisesti / Erittäin todennäköisesti*)

Survey questions in French

Q1: Les grands carnivores tels que les loups, les ours bruns et les lynx ont recolonisé certaines parties de l'Europe au cours des dernières décennies.D'une manière générale, diriez-vous que vous êtes favorable ou opposé au retour des grands carnivores ? (*Très opposé / Opposé / Sans opinion ou ne sais pas / Favorable / Très favorable*)

Q2: À mon avis, les populations de loups en Europe devraient être... (*Fortement réduites / Réduites / Maintenues comme elles sont / Augmentées / Fortement augmentées / Je ne sais pas*)

Q3: À mon avis, les populations de lynx en Europe devraient être... (*Fortement réduites / Réduites / Maintenues comme elles sont / Augmentées / Fortement augmentées / Je ne sais pas*)

Q4: À mon avis, les populations d'ours bruns en Europe devraient être... (*Fortement réduites / Réduites / Maintenues comme elles sont / Augmentées / Fortement augmentées / Je ne sais pas*)

Q5: De manière générale, diriez-vous que vous êtes favorable ou opposé à la chasse aux grands carnivores ? (*Très opposé / Opposé / Sans opinion ou ne sais pas / Favorable / Très favorable*)

Q6: Dans quelle mesure êtes-vous d'accord ou non avec les affirmations suivantes ?

1. Si un ours attaque une personne, cet ours doit être tué, quelles que soient les circonstances.
2. Les loups qui tuent du bétail devraient être tués.
3. Un lynx qui tue du bétail devrait être tué.

(*Tout à fait d'accord / Plutôt d'accord / Ni d'accord ni en désaccord / Plutôt en désaccord / Pas du tout d'accord*)

Q7: Quel est votre sexe ? (*Homme / Femme*)

Q8: Entrez l'année de votre naissance (au format AAAA)

Q9: Comment décririez-vous votre lieu de résidence actuelle ? (*Grande zone urbaine / Ville / Village / Maison isolée*)

Q10: En ce qui concerne la politique, veuillez indiquer laquelle des catégories suivantes vous considérez comme étant vous-même (*Extrême gauche / Gauche (socio-démocrate) / Centre / Droite (conservateur) / Extrême droite*)

Q11: Vous trouverez ci-dessous un certain nombre d'actions que vous pourriez entreprendre afin de soutenir la conservation des grands carnivores dans votre pays. Veuillez indiquer dans quelle mesure vous êtes susceptible ou non de... écrire ou appeler un politicien pour exprimer votre soutien aux efforts de conservation des carnivores (*Très peu probable / Assez peu probable / Indécis / Plutôt probable / Très probable*)

Q12: Vous trouverez ci-dessous un certain nombre d'actions que vous pourriez entreprendre afin de vous opposer aux populations de grands carnivores dans votre pays. Veuillez indiquer dans quelle mesure vous êtes susceptible ou non de... écrire ou appeler un politicien pour exprimer votre opposition aux efforts de conservation des carnivores (*Très peu probable / Assez peu probable / Indécis / Plutôt probable / Très probable*)

Survey questions in German

Q1: Große Raubtiere wie Wölfe, Braunbären und Luchse haben in den letzten Jahrzehnten Teile Europas wiederbesiedelt. Würden Sie generell sagen, dass Sie die Wiederansiedlung von großen Raubtieren unterstützen oder ablehnen? (*Lehne ich stark ab / Lehne ich ab / Neutral oder bin mir nicht sicher / Unterstütze ich / Unterstütze ich stark*)

Q2: Meiner Meinung nach sollte die Wolfpopulation in Europa … (*Stark reduziert werden / Reduziert werden / Gleich bleiben / Erhöht werden / Stark erhöht werden / Ich weiß nicht*)

Q3: Meiner Meinung nach sollte die Luchspopulation in Europa … (*Stark reduziert werden / Reduziert werden / Gleich bleiben / Erhöht werden / Stark erhöht werden / Ich weiß nicht*)

Q4: Meiner Meinung nach sollte die Braunbärenpopulation in Europa … (*Stark reduziert werden / Reduziert werden / Gleich bleiben / Erhöht werden / Stark erhöht werden / Ich weiß nicht*)

Q5: Würden Sie generell sagen, dass Sie die Jagd auf große Raubtiere befürworten oder ablehnen? (*Lehne ich stark ab / Lehne ich ab / Neutral oder bin mir nicht sicher / Unterstütze ich / Unterstütze ich stark*)

Q6: Inwieweit stimmen Sie den folgenden Aussagen zu oder nicht zu:

1. Wenn ein Bär einen Menschen angreift, sollte der Bär unabhängig von den Umständen getötet werden.
2. Wölfe, die Nutztiere töten, sollten getötet werden.
3. Luchse, die Nutztiere töten, sollten getötet werden.

(*Stimme voll und ganz zu / Stimme eher zu / Stimme weder zu noch nicht zu / Stimme eher nicht zu / Stimme überhaupt nicht zu*)

Q7: Was ist Ihr Geschlecht? (*Männlich / Weiblich*)

Q8: Geben Sie bitte Ihr Geburtsjahr (im Format JJJJ) an.

Q9: Wie würden Sie Ihren derzeitigen Wohnort oder Ihre Gemeinde beschreiben? (*Großes städtisches Gebiet / Stadt / Dorf / Abgeschiedenes Haus*)

Q10: Wenn es um Politik geht, geben Sie bitte an, zu welcher der folgenden Gruppen Sie sich selbst zählen. (*Linksextrem / Links (sozialdemokratisch) / Mitte / Rechts (konservativ) / Rechtsextrem*)

Q11: Nachfolgend sehen Sie eine Reihe von Maßnahmen, die Sie ergreifen können, um die Erhaltung von großen Raubtieren in Ihrem Land zu unterstützen. Geben Sie bitte an, wie wahrscheinlich oder unwahrscheinlich es ist, dass Sie ... Politikern schreiben oder anrufen, um Ihre Unterstützung für die Wiederansiedlung von Raubtieren zu bekunden. (*Sehr unwahrscheinlich / Eher unwahrscheinlich / Unentschlossen / Eher wahrscheinlich / Sehr wahrscheinlich*)

Q12: Nachfolgend sehen Sie eine Reihe von Maßnahmen, die Sie ergreifen können, um große Raubtiere in Ihrem Land zu bekämpfen. Geben Sie bitte an, wie wahrscheinlich oder unwahrscheinlich es ist, dass Sie ... Politikern schreiben oder anrufen, um Ihre Ablehnung gegen die Wiederansiedlung von Raubtieren zu bekunden. (*Sehr unwahrscheinlich / Eher unwahrscheinlich / Unentschlossen / Eher wahrscheinlich / Sehr wahrscheinlich*)

Survey questions in Greek

Q1: Μεγάλα σαρκοφάγα ζώα, όπως λύκοι, καφέ αρκούδες και λύγκες, αποικίζουν εκ νέου μέρη της Ευρώπης τις τελευταίες δεκαετίες. Σε γενικές γραμμές, θα λέγατε ότι είστε υπέρ ή κατά της πληθυσμιακής αποκατάστασης των μεγάλων σαρκοφάγων; (*Απολύτως κατά / Κατά / Ουδέτερη θέση ή δεν γνωρίζω / Υπέρ / Απολύτως υπέρ*)

Q2: Κατά τη γνώμη μου, οι πληθυσμοί λύκου στην Ευρώπη θα πρέπει… (*Να μειωθούν πολύ / Μειώθηκε / Να μείνουν περίπου οι ίδιοι / Αυξήθηκε / Να αυξηθούν πολύ / Δεν ξέρω*)

Q3: Κατά τη γνώμη μου, οι πληθυσμοί λύγκα στην Ευρώπη θα πρέπει… (*Να μειωθούν πολύ / Μειώθηκε / Να μείνουν περίπου οι ίδιοι / Αυξήθηκε / Να αυξηθούν πολύ / Δεν ξέρω*)

Q4: Κατά τη γνώμη μου, οι πληθυσμοί καφέ αρκούδας στην Ευρώπη θα πρέπει… (*Να μειωθούν πολύ / Μειώθηκε / Να μείνουν περίπου οι ίδιοι / Αυξήθηκε / Να αυξηθούν πολύ / Δεν ξέρω*)

Q5: Γενικά, θα λέγατε ότι είστε υπέρ ή κατά του κυνηγιού μεγάλων σαρκοφάγων; (*Απολύτως κατά / Κατά / Ουδέτερη θέση ή δεν γνωρίζω / Υπέρ / Απολύτως υπέρ*)

Q6: Σε ποιον βαθμό συμφωνείτε ή διαφωνείτε με τα ακόλουθα:

1. Εάν μια αρκούδα επιτεθεί σε κάποιον, αυτή η αρκούδα θα πρέπει να θανατωθεί, ανεξάρτητα από τις συνθήκες που έγινε η επίθεση.
2. Οι λύκοι που σκοτώνουν ζώα εκτροφής θα πρέπει να θανατώνονται.
3. Ένας λύγκας που σκοτώνει ζώα εκτροφής θα πρέπει να θανατώνεται.

(*Συμφωνώ απολύτως / Μάλλον συμφωνώ / Ούτε συμφωνώ, ούτε διαφωνώ / Μάλλον διαφωνώ / Διαφωνώ απολύτως*)

Q7: Ποιο είναι το φύλο σας; (*Άνδρας / Γυναίκα*)

Q8: Εισαγάγετε το έτος γέννησής σας (σε μορφή ΕΕΕΕ)

Q9: Πώς θα περιγράφατε την τρέχουσα κατοικία ή την κοινότητά σας; (*Μεγάλη αστική περιοχή / Πόλη / Χωριό / Απομονωμένο σπίτι*)

Q10: Όσον αφορά την πολιτική, υποδείξτε παρακάτω πού τοποθετείτε τον εαυτό σας. (*Άκρα αριστερά / Αριστερά (σοσιαλδημοκράτης) / Κέντρο / Δεξιά (συντηρητικός) / Άκρα δεξιά*)

Q11: Ακολουθούν ορισμένες ενέργειες που θα μπορούσατε να κάνετε για να υποστηρίξετε τη διατήρηση των μεγάλων σαρκοφάγων ζώων στη χώρα σας. Υποδείξτε πόσο πιθανό ή απίθανο είναι... Να γράψετε ή τηλεφωνήσετε σε κάποιον πολιτικό για να εκφράσετε την υποστήριξή σας στις προσπάθειες αποκατάστασης σαρκοφάγων ζώων. (*Πολύ απίθανο / Κάπως απίθανο / Δεν μπορώ να αποφασίσω / Κάπως πιθανό / Πολύ πιθανό*)

Q12: Ακολουθούν ορισμένες ενέργειες που θα μπορούσατε να κάνετε για να αντιταχθείτε στην ύπαρξη πληθυσμών μεγάλων σαρκοφάγων ζώων στη χώρα σας. Υποδείξτε πόσο πιθανό ή απίθανο είναι να... Να γράψετε ή τηλεφωνήσετε σε κάποιον πολιτικό για να εκφράσετε την αντίθεσή σας στις προσπάθειες αποκατάστασης σαρκοφάγων ζώων. (*Πολύ απίθανο / Κάπως απίθανο / Δεν μπορώ να αποφασίσω / Κάπως πιθανό / Πολύ πιθανό*)

Survey questions in Hungarian

Q1: A nagytestű ragadozók, például a farkasok, a barnamedvék és a hiúzok az elmúlt évtizedekben újra benépesítették Európa egyes részeit. Általánosságban azt mondaná, hogy támogatja vagy ellenzi a nagyragadozók állományának helyreállítását? (*Határozottan ellenzem / Ellenzem / Semleges vagyok vagy nem vagyok biztos benne / Támogatom / Határozottan támogatom*)

Q2: Véleményem szerint a farkaspopulációkkal Európában a következőt kellene csinálni: (*Nagymértékben csökkenteni / Csökkenteni / Nagyjából azonos mértékben tartani / Megnövelni / Nagyon megnövelni / Nem tudom*)

Q3: Véleményem szerint a hiúzpopulációkkal Európában a következőt kellene csinálni: (*Nagymértékben csökkenteni / Csökkenteni / Nagyjából azonos mértékben tartani / Megnövelni / Nagyon megnövelni / Nem tudom*)

Q4: Véleményem szerint a barnamedve-populációkkal Európában a következőt kellene csinálni: (*Nagymértékben csökkenteni / Csökkenteni / Nagyjából azonos mértékben tartani / Megnövelni / Nagyon megnövelni / Nem tudom*)

Q5: Általánosságban azt mondaná, hogy támogatja vagy ellenzi a nagyragadozók vadászatát? (*Határozottan ellenzem / Ellenzem / Semleges vagyok vagy nem vagyok biztos benne / Támogatom / Határozottan támogatom*)

Q6: Mennyire ért egyet vagy mennyire nem ért egyet a következőkkel:

1. Ha egy medve megtámad egy embert, azt a medvét a körülményektől függetlenül le kell ölni.
2. A jószágot ölő farkasokat le kell ölni.
3. A jószágot ölő hiúzokat le kell ölni.

(*Határozottan egyetértek / Részben egyetértek / Nem értek egyet, de nem is ellenzem / Részben nem értek egyet / Határozottan nem értek egyet*)

Q7: Milyen nemű Ön? (*Férfi / Nő*)

Q8: Adja meg a születési évét (ÉÉÉÉ formátumban)

Q9: Hogyan jellemezné jelenlegi lakóhelyét vagy közösségét? (*Nagyvárosi terület / Város / Falu / Elszigetelt ház*)

Q10: Ha politikáról van szó, kérjük, jelölje meg, hogy az alábbiak közül melyiknek tartja magát. (*Szélsőbaloldali / Baloldali (szociáldemokrata) / Centrista / Jobboldali (konzervatív) / Szélsőjobboldali*)

Q11: Az alábbiakban számos olyan intézkedést ismertetünk, amelyekkel Ön is támogathatja a nagyragadozók védelmét az Ön országában. Kérjük, jelezze, mennyire valószínű vagy valószínűtlen, hogy... Ír vagy telefonál egy politikusnak, hogy kifejezze támogatását a ragadozóállomány helyreállítására irányuló erőfeszítésekhez. (*Nagyon valószínűtlen / Valamennyire valószínűtlen / Semleges állásponton vagyok / Valamennyire valószínű / Nagyon valószínű*)

Q12: Az alábbiakban számos olyan intézkedést ismertetünk, amelyekkel Ön is felléphet a nagyragadozó populációk ellen az Ön országában. Kérjük, jelezze, mennyire valószínű vagy valószínűtlen, hogy... Ír vagy telefonál egy politikusnak, hogy kifejezze ellenérzését a ragadozóállomány helyreállítására irányuló erőfeszítésekhez. (*Nagyon valószínűtlen / Valamennyire valószínűtlen / Semleges állásponton vagyok / Valamennyire valószínű / Nagyon valószínű*)

Survey questions in Italian

Q1: Negli ultimi decenni, grandi carnivori come il lupo, l'orso bruno e la lince hanno ricolonizzato parte dell'Europa.In generale, è favorevole o contrario al recupero dei grandi carnivori? (*Fortemente contrario / Contrario / Neutrale o indeciso / Favorevole / Fortemente favorevole*)

Q2: A mio parere, le popolazioni di lupi in Europa dovrebbero... (*Diminuire notevolmente / Diminuire / Rimanere più o meno le stesse / Aumentare / Aumentare notevolmente / Non lo so*)

Q3: A mio parere, le popolazioni di linci in Europa dovrebbero... (*Diminuire notevolmente / Diminuire / Rimanere più o meno le stesse / Aumentare / Aumentare notevolmente / Non lo so*)

Q4: A mio parere, le popolazioni di orso bruno in Europa dovrebbero... (*Diminuire notevolmente / Diminuire / Rimanere più o meno le stesse / Aumentare / Aumentare notevolmente / Non lo so*)

Q5: In generale, è favorevole o contrario alla caccia ai grandi carnivori? (*Fortemente contrario / Contrario / Neutrale o indeciso / Favorevole / Fortemente favorevole*)

Q6: In che misura è d'accordo o in disaccordo con quanto segue:

1. Se un orso attacca una persona, deve essere ucciso a prescindere dalle circostanze.
2. I lupi che uccidono il bestiame dovrebbero essere uccisi.
3. Una lince che uccide il bestiame dovrebbe essere uccisa.

(*Fortemente d'accordo / D'accordo / Né d'accordo né in disaccordo / In disaccordo / Fortemente in disaccordo*)

Q7: Qual è il suo sesso? (*Maschio / Femmina*)

Q8: Inserire l'anno di nascita (nel formato AAAA)

Q9: Come descriverebbe il suo attuale luogo di residenza? (*Grande area urbana / Città / Villaggio / Casa isolata*)

Q10: Quando si parla di politica, la preghiamo di indicare quale dei seguenti si considera. (*Estrema sinistra / Sinistra (socialdemocratica) / Centro / Destra (conservatrice) / Estrema destra*)

Q11: Di seguito sono elencate alcune azioni che si potrebbero intraprendere per sostenere la conservazione dei grandi carnivori nel vostro Paese. Indicare la probabilità che lei possa effettivamente... …scrivere o chiamare un politico per esprimere il vostro sostegno al ripristino dei grandi carnivori. (*Molto improbabile / Alquanto improbabile / Indecisi / Abbastanza probabile / Molto probabile*)

Q12: Di seguito sono elencate alcune azioni da poter intraprendere per opporsi alla conservazione delle popolazioni di grandi carnivori nel vostro Paese. Indicare la probabilità che lei possa effettivamente... …scrivere o telefonare a un politico per esprimere la vostra opposizione al ripristino dei grandi carnivori. (*Molto improbabile / Alquanto improbabile / Indecisi / Abbastanza probabile / Molto probabile*)

Survey questions in Latvian

Q1: Lielie plēsēji, piemēram, vilki, brūnie lāči un lūši, pēdējās desmitgadēs ir rekolonizējuši dažas Eiropas daļas. Vai kopumā atbalstāt vai esat pret lielo plēsēju populācijas atjaunošanos? (*Esmu noteikti pret / Esmu pret / Neitrāla attieksme vai nezinu atbildi / Atbalstu / Noteikti atbalstu*)

Q2: Manuprāt, vilku populācija Eiropā … (*Ievērojami jāsamazina / Jāsamazina / Jāsaglabā esošajā līmenī / Jāpalielina / Ievērojami jāpalielina / Es nezinu*)

Q3: Manuprāt, lūšu populācija Eiropā … (*Ievērojami jāsamazina / Jāsamazina / Jāsaglabā esošajā līmenī / Jāpalielina / Ievērojami jāpalielina / Es nezinu*)

Q4: Manuprāt, brūno lāču populācija Eiropā … (*Ievērojami jāsamazina / Jāsamazina / Jāsaglabā esošajā līmenī / Jāpalielina / Ievērojami jāpalielina / Es nezinu*)

Q5: Vai kopumā atbalstāt vai esat pret lielo plēsēju medībām? (*Esmu noteikti pret / Esmu pret / Neitrāla attieksme vai nezinu atbildi / Atbalstu / Noteikti atbalstu*)

Q6: Cik lielā mērā piekrītat vai nepiekrītat tālāk minētajiem apgalvojumiem:

1. Ja lācis uzbrūk cilvēkam, šis lācis jānogalina neatkarīgi no apstākļiem.
2. Vilki, kas nogalina mājlopus, ir jānogalina.
3. Lūši, kas nogalina mājlopus, ir jānogalina.

(*Pilnībā piekrītu / Nedaudz piekrītu / Ne piekrītu, ne nepiekrītu / Nedaudz piekrītu / Pilnībā nepiekrītu*)

Q7: Kāds ir Jūsu dzimums? (*Vīrietis / Sieviete*)

Q8: Ievadiet savu dzimšanas gadu (GGGG formātā)

Q9: Kā Jūs aprakstītu savu pašreizējo dzīvesvietu vai kopienu? (*Liels pilsētas rajons / Pilsēta / Ciemats / Atsevišķa māja*)

Q10: Domājot par politiku, par kuru no tālāk minētajiem uzskatāt sevi: (*Galēji kreisais / Kreisais (sociāldemokrāts) / Centrists / Labējais (konservatīvais) / Galēji labējais*)

Q11: Tālāk minētas vairākas darbības, kuras varat veikt, lai atbalstītu lielo plēsēju aizsardzību savā valstī. Lūdzu, norādiet, cik liela vai maza ir iespējamība, ka Jūs... Uzrakstīsiet vai piezvanīsiet politiķim, lai paustu atbalstu centieniem lielo plēsēju populācijas atjaunošanā. (*Ļoti maza / Diezgan maza / Neesmu izlēmis(-usi) / Diezgan liela iespējamība / Ļoti iespējams*)

Q12: Tālāk minētas vairākas darbības, kuras varat veikt, lai iestātos pret lielo plēsēju populāciju Jūsu valstī. Lūdzu, norādiet, cik liela vai maza ir iespējamība, ka Jūs... Uzrakstīsiet vai piezvanīsiet politiķim, lai iestātos pret centieniem lielo plēsēju populācijas atjaunošanā. (*Ļoti maza / Diezgan maza / Neesmu izlēmis(-usi) / Diezgan liela iespējamība / Ļoti iespējams*)

Survey questions in Lithuanian

Q1: Stambūs plėšrūnai, tokie kaip vilkai, rudieji lokiai ir lūšys, pastaraisiais dešimtmečiais iš naujo paplito kai kuriose Europos teritorijose. Ar jūs bendrai pritariate stambiųjų plėšrūnų populiacijos atkūrimui? (*Labai prieštarauju / Prieštarauju / Neturiu nuomonės arba nesu tikras / Pritariu / Labai pritariu*)

Q2: Mano nuomone, vilkų populiacija Europoje turėtų būti… (*Labai sumažinta / Sumažinta / Likti daugmaž tokia pat / Padidinta / Labai padidinta / Nežinau*)

Q3: Mano nuomone, lūšių populiacija Europoje turėtų būti… (*Labai sumažinta / Sumažinta / Likti daugmaž tokia pat / Padidinta / Labai padidinta / Nežinau*)

Q4: Mano nuomone, rudųjų meškų populiacija Europoje turėtų būti… (*Labai sumažinta / Sumažinta / Likti daugmaž tokia pat / Padidinta / Labai padidinta / Nežinau*)

Q5: Ar jūs bendrai pritariate stambiųjų plėšrūnų medžioklei? (*Labai prieštarauju / Prieštarauju / Neturiu nuomonės arba nesu tikras / Pritariu / Labai pritariu*)

Q6: Kiek sutinkate su šiais teiginiais:

1. Jei lokys užpuola žmogų, lokį reikia nužudyti nepaisant aplinkybių.
2. Vilkai, kurie pjauna gyvulius, turi būti nužudyti.
3. Lūšys, kurios pjauna gyvulius, turi būti nužudytos.

(*Labai sutinku / Iš dalies sutinku / Nei sutinku, nei nesutinku / Iš dalies nesutinku / Labai nesutinku*)

Q7: Kokia jūsų lytis? (*Vyras / Moteris*)

Q8: Įveskite savo gimimo metus (MMMM formatu)

Q9: Kur gyvenate? (*Didmiestis / Miestas / Kaimas / Vienkiemis*)

Q10: Kalbant apie politiką, nurodykite, prie kurių pažiūrų priskirtumėte save. (*Ekstremalūs kairieji / Kairieji (socialdemokratai) / Centristai / Dešinieji (konservatoriai) / Ekstremalūs dešinieji*)

Q11: Toliau nurodyti veiksmai, kurių galėtumėte imtis, kad palaikytumėte stambiųjų plėšrūnų apsaugą savo šalyje.Nurodykite, kiek tikėtina, kad... Parašysite ar paskambinsite politikui, kad išreikštumėte savo paramą plėšrūnų populiacijos atkūrimo pastangoms. (*Mažai tikėtina / Nelabai tikėtina / Neapsisprendžiu / Šiek tiek tikėtina / Labai tikėtina*)

Q12: Toliau pateikiami veiksmai, kurių galėtumėte imtis, kad pasipriešintumėte didelėms plėšrūnų populiacijoms savo šalyje.Nurodykite, kiek tikėtina, kad... Parašysite ar paskambinsite politikui, kad išreikštumėte savo prieštaravimą plėšrūnų populiacijos atkūrimo pastangoms. (*Mažai tikėtina / Nelabai tikėtina / Nežinau / Šiek tiek tikėtina / Labai tikėtina*)

Survey questions in Polish

Q1: Duże zwierzęta mięsożerne, takie jak wilki, niedźwiedzie brunatne i rysie, w ostatnich dziesięcioleciach rekolonizują część Europy.Ogólnie rzecz biorąc, czy powiedziałbyś że popierasz czy sprzeciwiasz się przywróceniu (restytucji) dużych zwierząt mięsożernych? (*Zdecydowanie się sprzeciwiam / Sprzeciwiam się / Jestem neutralny lub nie wiem / Popieram / Zdecydowanie popieram*)

Q2: Moim zdaniem populacje wilków w Europie powinny być... (*Znacznie zmniejszone / Zmniejszone / Pozostać na tym samym poziomie / Zwiększone / Znacznie zwiększone / Nie wiem*)

Q3: Moim zdaniem, populacje rysia w Europie powinny być... (*Znacznie zmniejszone / Zmniejszone / Pozostać na tym samym poziomie / Zwiększone / Znacznie zwiększone / Nie wiem*)

Q4: Moim zdaniem populacje niedźwiedzi brunatnych w Europie powinny być... (*Znacznie zmniejszone / Zmniejszone / Pozostać na tym samym poziomie / Zwiększone / Znacznie zwiększone / Nie wiem*)

Q5: Ogólnie rzecz biorąc, czy powiedziałbyś, że popierasz lub sprzeciwiasz się polowaniu na duże zwierzęta mięsożerne? (*Zdecydowanie się sprzeciwiam / Sprzeciwiam się / Jestem neutralny lub nie wiem / Popieram / Zdecydowanie popieram*)

Q6: W jakim stopniu zgadzasz się lub nie zgadzasz się z następującymi kwestiami:

1. Jeśli niedźwiedź zaatakuje człowieka, należy go zabić bez względu na okoliczności.
2. Wilki, które zabijają zwierzęta gospodarskie, powinny być zabijane.
3. Ryś, który zabija zwierzęta gospodarskie powinien zostać zabity.

(*Zdecydowanie zgadzam się / W pewnym stopniu zgadzam się / Ani się zgadzam, ani nie zgadzam / W pewnym stopniu nie zgadzam się / Zdecydowanie nie zgadzam się*)

Q7: Jaka jest Twoja płeć? (*Mężczyzna / Kobieta*)

Q8: Wpisz rok swojego urodzenia (w formacie rrrr)

Q9: Jak opisałbyś swoje obecne miejsce zamieszkania? (*Duży obszar miejski / Miasto / Wieś / Dom wolno stojący poza obszarem zabudowanym*)

Q10: Jeśli chodzi o politykę, proszę wskazać, do której z poniższych opcji siebie zaliczasz. (*Skrajna lewica / Lewica (socjaldemokratyczna) / Centrum / Prawica (konserwatywna) / Skrajna prawica*)

Q11: Poniżej podano szereg działań, które mógłbyś podjąć w celu wsparcia ochrony dużych drapieżników w Twoim kraju. Proszę wskazać, jak bardzo prawdopodobne lub mało prawdopodobne jest, że... Napiszesz lub zadzwonisz do polityka, aby wyrazić swoje poparcie dla działań na rzecz restytucji drapieżników. (*Bardzo mało prawdopodobne / Mało prawdopodobne / Trudno powiedzieć / Dosyć prawdopodobne / Bardzo prawdopodobne*)

Q12: Poniżej podano szereg działań, które mógłbyś podjąć w celu przeciwstawienia się dużym populacjom drapieżników w Twoim kraju. Proszę wskazać, jak bardzo prawdopodobne lub mało prawdopodobne jest, że... Napiszesz lub zadzwonisz do polityka, aby wyrazić swój sprzeciw wobec działań na rzecz restytucji drapieżników. (*Bardzo mało prawdopodobne / Mało prawdopodobne / Trudno powiedzieć / Dosyć prawdopodobne / Bardzo prawdopodobne*)

Survey questions in Portuguese

Q1: Nas últimas décadas, os grandes carnívoros, como os lobos, ursos pardos e linces começaram a repovoar certas partes da Europa. De uma forma geral, diria que apoia ou que se opõe à recuperação dos grandes carnívoros? (*Oponho-me totalmente / Oponho-me / Tenho uma opinião neutra ou não tenho a certeza / Apoio / Apoio totalmente*)

Q2: Na minha opinião, as populações de lobos na Europa deveriam ser… (*Muito reduzidas / Reduzidas / Manter-se praticamente idênticas / Aumentadas / Muito aumentadas / Não sei*)

Q3: Na minha opinião, as populações de linces na Europa deveriam ser… (*Muito reduzidas / Reduzidas / Manter-se praticamente idênticas / Aumentadas / Muito aumentadas / Não sei*)

Q4: Na minha opinião, as populações de ursos pardos na Europa deveriam ser… (*Muito reduzidas / Reduzidas / Manter-se praticamente idênticas / Aumentadas / Muito aumentadas / Não sei*)

Q5: De uma forma geral, diria que apoia ou que se opõe à caça de grandes carnívoros? (*Oponho-me totalmente / Oponho-me / Tenho uma opinião neutra ou não tenho a certeza / Apoio / Apoio totalmente*)

Q6: Até que ponto concorda com ou discorda das seguintes afirmações:

1. Se um urso atacar uma pessoa, esse urso deverá ser abatido, independentemente das circunstâncias.
2. Lobos que matem gado deveriam ser abatidos.
3. Um lince que mate gado deveria ser abatido.

(*Concordo totalmente / Concordo parcialmente / Não concordo nem discordo / Discordo parcialmente / Discordo totalmente*)

Q7: Como descreveria o seu género? (*Masculino / Feminino*)

Q8: Introduza o ano em que nasceu (num formato AAAA)

Q9: Como descreveria a sua área de residência ou comunidade local? (*Grande zona urbana / Cidade / Vila / Moradia isolada*)

Q10: Qual das seguintes opções melhor descreveria o seu posicionamento político? (*Extrema esquerda / Esquerda (social democrata) / Centro / Direita (conservador) / Extrema direita*)

Q11: Abaixo encontrará algumas ações que poderia executar para apoiar a conservação de grandes carnívoros no seu país.Indique até que ponto é provável ou improvável que… Contacte por escrito ou telefone a um político para expressar o seu apoio para ações de recuperação de carnívoros. (*Muito improvável / Pouco improvável / Ainda não decidi / Pouco provável / Muito provável*)

Q12: Abaixo encontrará algumas ações que poderia executar para se opor à permanência de grandes populações de carnívoros no seu país.Indique até que ponto é provável ou improvável que… Contacte por escrito ou telefone a um político para expressar a sua oposição a ações de recuperação de carnívoros. (*Muito improvável / Pouco improvável / Ainda não decidi / Pouco provável / Muito provável*)

Survey questions in Romanian

Q1: Carnivorele mari, cum ar fi lupii, urșii bruni și râșii, au recolonizat unele părți ale Europei în ultimele decenii. În general, ați spune că sprijiniți sau vă opuneți refacerii populației de carnivore mari? (*Mă opun cu putere / Mă opun / Părere neutră sau nu sunt sigur(ă) / Susțin / Susțin cu putere*)

Q2: În opinia mea, populațiile de lupi din Europa ar trebui să fie… (*Rărite mult / Rărite / Păstrate aproximativ la fel / Crescute / Crescute mult / Nu știu*)

Q3: În opinia mea, populațiile de râși din Europa ar trebui să fie… (*Rărite mult / Rărite / Păstrate aproximativ la fel / Crescute / Crescute mult / Nu știu*)

Q4: În opinia mea, populațiile de urși bruni din Europa ar trebui să fie… (*Rărite mult / Rărite / Păstrate aproximativ la fel / Crescute / Crescute mult / Nu știu*)

Q5: În general, ați spune că sprijiniți sau vă opuneți vânătorii de carnivore mari? (*Mă opun cu putere / Mă opun / Părere neutră sau nu sunt sigur(ă) / Susțin / Susțin cu putere*)

Q6: În ce măsură sunteți sau nu sunteți de acord cu următoarele afirmații?
1. Dacă un urs atacă o persoană, acel urs ar trebui să fie ucis, indiferent de circumstanțe.
2. Lupii care ucid animale domestice ar trebui să fie uciși.
3. Un râs care ucide animale domestice ar trebui să fie ucis.

(*Acord total / Acord ușor / Nici acord, nici dezacord / Dezacord ușor / Dezacord total*)

Q7: Care este sexul dvs.? (*Bărbat / Femeie*)

Q8: Introduceți anul în care v-ați născut (în format AAAA)

Q9: Cum v-ați descrie actuala reședință sau comunitate? (*Zonă urbană mare / Oraș / Sat / Casă izolată*)

Q10: Când vine vorba de politică, vă rugăm să indicați cu care dintre următoarele politici vă identificați. (*Extremă stânga / Stânga (social-democrat) / Centru / Dreapta (conservator) / Extremă dreapta*)

Q11: Mai jos este prezentată o serie de acțiuni pe care le-ați putea realiza pentru a sprijini conservarea carnivorelor mari în țara dvs. Vă rugăm să indicați cât de probabil sau de improbabil este ca dvs. să... Îi scrieți unui politician sau să-l sunați pentru a vă exprima sprijinul pentru eforturile de refacere a populațiilor de carnivore. (*Foarte improbabil / Oarecum improbabil / Sunt indecis(ă) / Oarecum probabil / Foarte probabil*)

Q12: Mai jos este prezentată o serie de acțiuni pe care le-ați putea realiza pentru a vă opune conservării carnivorelor mari în țara dvs. Vă rugăm să indicați cât de probabil sau de improbabil este ca dvs. să... Îi scrieți unui politician sau să-l sunați pentru a vă exprima opoziția față de eforturile de refacere a populațiilor de carnivore. (*Foarte improbabil / Oarecum improbabil / Sunt indecis(ă) / Oarecum probabil / Foarte probabil*)

Survey questions in Slovakian

Q1: Veľké mäsožravce ako vlky, medvede hnedé a rysy v posledných desaťročiach rekolonizujú časti Európy. Povedali by ste vo všeobecnej rovine, že podporujete alebo ste proti obnove veľkých mäsožravcov? (*Som zásadne proti / Som proti / Som neutrálny alebo si nie som istý/á / Podporujem / Silne podporujem*)

Q2: Podľa mňa populácia vlkov v Európe by mala byť… (*Výrazne menšia / Menšia / Zostať približne rovnaká / Väčšia / Výrazne väčšia / Neviem*)

Q3: Podľa mňa populácia rysov v Európe by mala byť… (*Výrazne menšia / Menšia / Zostať približne rovnaká / Väčšia / Výrazne väčšia / Neviem*)

Q4: Podľa mňa populácia medveďa hnedého v Európe by mala byť… (*Výrazne menšia / Menšia / Zostať približne rovnaká / Väčšia / Výrazne väčšia / Neviem*)

Q5: Povedali by ste vo všeobecnosti, že podporujete alebo ste proti lovu veľkých mäsožravcov? (*Som zásadne proti / Som proti / Som neutrálny alebo si nie som istý/á / Podporujem / Silne podporujem*)

Q6: Do akej miery súhlasíte alebo nesúhlasíte s nasledujúcim výrokom:

1. Ak medveď napadne človeka, mal by byť zabitý bez ohľadu na okolnosti.
2. Vlci, ktorí zabíjajú hospodárske zvieratá, by mali byť zabití.
3. Rys, ktorý zabíja hospodárske zvieratá, by mal byť zabitý.

(*Veľmi súhlasím, Do istej miery súhlasím, Ani súhlasím, ani nesúhlasím, Do istej miery nesúhlasím, Veľmi nesúhlasím*)

Q7: Aké je vaše pohlavie? (*Muž / Žena*)

Q8: Zadajte rok, v ktorom ste sa narodili (vo formáte YYYY)

Q9: Ako by ste opísali svoje súčasné bydlisko alebo komunitu? (*Veľká mestská oblasť / Mesto / Obec / Izolovaný dom*)

Q10: Pokiaľ ide o politiku, uveďte, s ktorou z nasledujúcich možností sa stotožňujete. (*Extrémna ľavica / Ľavica (sociálny demokrat) / Stred / Pravica (konzervatívna) / Krajná pravica*)

Q11: Nižšie uvádzame niekoľko aktivít, ktoré môžete vykonať na podporu ochrany veľkých mäsožravcov vo vašej krajine. Uveďte, prosím, nakoľko je pravdepodobné alebo nepravdepodobné, že... Napíšete alebo zavoláte politikovi, aby ste vyjadrili svoju podporu úsiliu o obnovu mäsožravcov. (*Veľmi nepravdepodobné / Trochu nepravdepodobné / Nie som rozhodnutý/á / Je to trochu pravdepodobné / Je to veľmi pravdepodobné*)

Q12: Nižšie uvádzame niekoľko aktivít, ktoré môžete vykonať na zabránenie vzniku veľkej populácie mäsožravcov vo vašej krajine. Uveďte, prosím, nakoľko je pravdepodobné alebo nepravdepodobné, že... Napíšete alebo zavoláte politikovi, aby ste vyjadrili svoj nesúhlas s úsilím o obnovu mäsožravcov. (*Veľmi nepravdepodobné / Trochu nepravdepodobné / Nie som rozhodnutý/á / Je to trochu pravdepodobné / Je to veľmi pravdepodobné*)

Survey questions in Slovenian

Q1: Velike zveri, kot so volkovi, rjavi medvedi in risi, v zadnjih desetletjih ponovno naseljujejo dele Evrope. Bi na splošno rekli, da podpirate okrevanje populacije velikih zveri ali mu nasprotujete? (*Odločno nasprotujem / Nasprotujem / Nisem opredeljen/-a ali nisem prepričan/-a / Podpiram / Močno podpiram*)

Q2: Po mojem mnenju bi se populacije volkov v Evropi morale … (*Zelo zmanjšati / Zmanjšati / Ohraniti približno enake / Povečati / Močno povečati / Ne vem*)

Q3: Po mojem mnenju bi se populacije risov v Evropi morale … (*Zelo zmanjšati / Zmanjšati / Ohraniti približno enake / Povečati / Močno povečati / Ne vem*)

Q4: Po mojem mnenju bi se populacije rjavega medveda v Evropi morale … (*Zelo zmanjšati / Zmanjšati / Ohraniti približno enake / Povečati / Močno povečati / Ne vem*)

Q5: Bi na splošno rekli, da podpirate lov na velike zveri ali mu nasprotujete? (*Odločno nasprotujem / Nasprotujem / Nisem opredeljen/-a ali nisem prepričan/-a / Podpiram / Močno podpiram*)

Q6: V kolikšni meri se strinjate ali ne strinjate z naslednjim:
1. Če medved napade človeka, ga je treba usmrtiti ne glede na okoliščine.
2. Volkove, ki pobijajo živino, je treba usmrtiti.
3. Risa, ki ubija živino, je treba usmrtiti.

(*Popolnoma se strinjam / Deloma se strinjam / Niti se strinjam niti se ne strinjam / Deloma se ne strinjam / Nikakor se ne strinjam*)

Q7: Katerega spola ste? (*Moški / Ženska*)

Q8: Vnesite leto rojstva (v obliki LLLL)

Q9: Kako bi opisali svoje trenutno prebivališče ali skupnost? (*Večje mestno območje / (Vele)mesto / Vas / Hiša na samem*)

Q10: Navedite svojo politično pripadnost. (*Skrajna levica / Levica (socialna demokracija) / Sredinska politika / Desnica (konservatizem) / Skrajna desnica*)

Q11: Spodaj je nekaj dejanj, ki jih lahko izvedete, da podprete ohranjanje velikih zveri v svoji državi. Prosimo, da navedete, kako verjetno ali malo verjetno je, da ... Pišete politiku ali ga pokličete, da izrazite svojo podporo prizadevanjem za obnovo populacije zveri. (*Zelo malo verjetno / Nekoliko neverjetno / Neopredeljeno / Nekoliko verjetno / Zelo verjetno*)

Q12: Spodaj je nekaj dejanj, ki jih lahko izvedete, da nasprotujete velikim populacijam zveri v svoji državi. Prosimo, da navedete, kako verjetno ali malo verjetno je, da ... Pišete politiku ali ga pokličete, da izrazite svoje nasprotovanje prizadevanjem za obnovo populacije zveri. (*Zelo malo verjetno / Nekoliko neverjetno / Neopredeljeno / Nekoliko verjetno / Zelo verjetno*)

Survey questions in Spanish

Q1: En las últimas décadas, especies de grandes carnívoros como lobos, osos pardos y linces han vuelto a recolonizar partes de Europa.En términos generales, ¿diría que apoya o se opone a la recuperación de los grandes carnívoros? (*Totalmente en contra / En contra / Neutral o no estoy seguro / A favor / Totalmente a favor*)

Q2: En mi opinión, las poblaciones de lobos en Europa deberían... (*Disminuir considerablemente / Disminuir / Mantenerse más o menos igual / Aumentar / Aumentar considerablemente / No lo sé*)

Q3: En mi opinión, las poblaciones de lince en Europa deberían... (*Disminuir considerablemente / Disminuir / Mantenerse más o menos igual / Aumentar / Aumentar considerablemente / No lo sé*)

Q4: En mi opinión, las poblaciones de oso pardo en Europa deberían... (*Disminuir considerablemente / Disminuir / Mantenerse más o menos igual / Aumentar / Aumentar considerablemente / No lo sé*)

Q5: En general, ¿diría que apoya o se opone a la caza de grandes carnívoros? (*Totalmente en contra / En contra / Neutral o no estoy seguro / A favor / Totalmente a favor*)

Q6: ¿En qué medida está de acuerdo o en desacuerdo con lo siguiente?
1. Si un oso ataca a una persona, debe ser sacrificado independientemente de las circunstancias.
2. Los lobos que matan al ganado deben ser sacrificados.
3. Un lince que mate ganado debe ser sacrificado.

(*Totalmente de acuerdo / Algo de acuerdo / Ni de acuerdo ni en desacuerdo / Algo en desacuerdo / Totalmente en desacuerdo*)

Q7: ¿Cuál es su sexo? (*Masculino / Femenino*)

Q8: Introduzca su año de nacimiento (en formato AAAA)

Q9:¿Cómo describiría su lugar de residencia? (*Área urbana/metropolitana / Ciudad / Pueblo / Casa aislada*)

Q10: En lo que respecta a la política, indique cuál de las siguientes categorías se considera usted. (*Extrema izquierda / Izquierda (socialdemócrata) / Centro / Derecha (conservador) / Extrema derecha*)

Q11: A continuación, se enumeran una serie de acciones que podría llevar a cabo para apoyar la conservación de los grandes carnívoros en su país. Por favor, indique si es probable o improbable que... Escriba o llame a un político para expresar su apoyo a los esfuerzos de recuperación de carnívoros. (*Muy improbable / Algo improbable / Indeciso / Algo probable / Muy probable*)

Q12: A continuación se indican una serie de acciones que podría llevar a cabo para oponerse a las poblaciones de grandes carnívoros en su país. Indique si es probable o improbable que... Escriba o llame a un político para expresar su oposición a los esfuerzos de recuperación de carnívoros. (*Muy improbable / Algo improbable / Indecisos / Algo probable / Muy probable*)

Survey questions in Swedish

Q1: Stora rovdjur som varg, brunbjörn och lodjur har återkoloniserat delar av Europa under de senaste årtiondena.Skulle du generellt sett säga att du stödjer eller motsätter dig att de stora rovdjuren återetablerar sig? (*Starkt emot / Delvis emot / Neutral eller osäker / Delvis för / Starkt för*)

Q2: Jag anser att vargpopulationerna i Europa bör... (*Minska kraftigt / Minska / Förbli ungefär oförändrade / Öka / Öka kraftigt / Jag vet inte*)

Q3: Jag anser att lodjurspopulationerna i Europa bör... (*Minska kraftigt / Minska / Förbli ungefär oförändrade / Öka / Öka kraftigt / Jag vet inte*)

Q4: Jag anser att brunbjörnspopulationerna i Europa bör... (*Minska kraftigt / Minska / Förbli ungefär oförändrade / Öka / Öka kraftigt / Jag vet inte*)

Q5: Skulle du generellt sett säga att du stöder eller motsätter dig jakt på stora rovdjur? (*Starkt emot / Delvis emot / Neutral eller osäker / Delvis för / Starkt för*)

Q6: I vilken utsträckning håller du med eller inte håller med om följande:

1. Om en björn attackerar en person ska björnen dödas oavsett omständigheterna.
2. Vargar som dödar tamdjur bör avlivas.
3. Ett lodjur som dödar tamdjur bör avlivas.

(*Instämmer helt / Instämmer delvis / Varken eller / Delvis oenig / Helt oenig*)

Q7: Vilket är ditt kön? (*Man / Kvinna*)

Q8: Ange ditt födelseår (i formatet ÅÅÅÅ)

Q9: Hur skulle du beskriva din nuvarande bostadort? (*Storstadsområde / Stad / Mindre ort / Landsbygd)*

Q10: När det gäller politik, ange vilken av följande inriktningar du anser dig vara: (*Extremt vänster / Socialdemokrat / Mitten / Konservativ / Extremt höger*)

Q11: Nedan finns ett antal åtgärder som du kan vidta för att stödja bevarandet av stora rovdjur i ditt land. Ange hur troligt eller osannolikt det är att du kommer att... Skriva eller ringa till en politiker för att uttrycka ditt stöd för arbetet med att få tillbaka rovdjur. (*Mycket osannolikt / Ganska osannolikt / Vet inte / Ganska troligt / Mycket troligt*)

Q12: Nedan följer ett antal åtgärder som du skulle kunna vidta för att motverka populationer av stora rovdjur i ditt land. Ange hur troligt eller osannolikt det är att du kommer att... Skriva eller ringa till en politiker för att uttrycka ditt motstånd mot åtgärder för att återställa rovdjur. (*Mycket osannolikt / Ganska osannolikt / Vet inte / Ganska troligt / Mycket troligt*)

Supplementary References

1. Freese, J. & Jin, O. Online Nonprobability Samples. *Annual Review of Sociology* **51**, 109–128 (2025).
2. Lehdonvirta, V., Oksanen, A., Räsänen, P. & Blank, G. Social Media, Web, and Panel Surveys: Using Non-Probability Samples in Social and Policy Research. *Policy & Internet* **13**, 134–155 (2021).
3. Vaske, J. J., Don Carlos, A. W., Manfredo, M. J. & Teel, T. L. Evaluating alternative survey methodologies in human dimensions of wildlife research. *Human Dimensions of Wildlife* **28**, 320–334 (2023).
4. Daikeler, J., Silber, H. & Bošnjak, M. A Meta-Analysis of How Country-Level Factors Affect Web Survey Response Rates. *International Journal of Market Research* **64**, 306–333 (2022).
5. Statistisches Bundesamt. Population by nationaly and federal states. *Federal Statistical Office* https://www.destatis.de/EN/Themes/Society-Environment/Population/Current-Population/Tables/population-by-laender.html (2024).
6. Biermann, P. & Welsch, H. *Changing Conditions, Persistent Mentality: An Anatomy of East German Unhappiness, 1990-2016*. https://www.econstor.eu/handle/10419/196145 (2019).
7. Kountouris, Y. Do political systems have a lasting effect on climate change concern? Evidence from Germany after reunification. *Environmental Research Letters* **16**, 074040 (2021).
8. van Hoorn, A. & Maseland, R. Cultural differences between East and West Germany after 1991: Communist values versus economic performance? *Journal of Economic Behavior & Organization* **76**, 791–804 (2010).
9. Eurostat. Statistics | Eurostat: Median age by sex for 2021. https://doi.org/10.2908/EQ_POP04 (2024).
10. Eurostat. Urban-rural Europe - introduction. https://ec.europa.eu/eurostat/statistics-explained/index.php?title=Urban-rural_Europe_-_introduction (2024).
