# Supplementary material for: Europeans support large carnivore recovery while opposing both further population growth and hunting
Source: Nat Ecol Evol. 2025 Nov 25;10(1):28–33. doi: 10.1038/s41559-025-02914-1 (PMC12789005; doi:10.1038/s41559-025-02914-1)
Supplement: Supplementary file 2 — Reporting Summary [file 41559_2025_2914_MOESM2_ESM.pdf]

nature portfolio

Corresponding author(s): Guillaume Chapron

Last updated by author(s): Oct 10, 2025

Reporting Summary

Nature Portfolio wishes to improve the reproducibility of the work that we publish. This form provides structure for consistency and transparency in reporting. For further information on Nature Portfolio policies, see our Editorial Policies and the Editorial Policy Checklist.

Statistics

For all statistical analyses, confirm that the following items are present in the figure legend, table legend, main text, or Methods section.

n/a	Confirmed	
☐	☒	The exact sample size (*n*) for each experimental group/condition, given as a discrete number and unit of measurement
☒	☐	A statement on whether measurements were taken from distinct samples or whether the same sample was measured repeatedly
☒	☐	The statistical test(s) used AND whether they are one- or two-sided<br>*Only common tests should be described solely by name; describe more complex techniques in the Methods section.*
☒	☐	A description of all covariates tested
☒	☐	A description of any assumptions or corrections, such as tests of normality and adjustment for multiple comparisons
☐	☒	A full description of the statistical parameters including central tendency (e.g. means) or other basic estimates (e.g. regression coefficient) AND variation (e.g. standard deviation) or associated estimates of uncertainty (e.g. confidence intervals)
☒	☐	For null hypothesis testing, the test statistic (e.g. *F*, *t*, *r*) with confidence intervals, effect sizes, degrees of freedom and *P* value noted<br>*Give P values as exact values whenever suitable.*
☐	☒	For Bayesian analysis, information on the choice of priors and Markov chain Monte Carlo settings
☐	☒	For hierarchical and complex designs, identification of the appropriate level for tests and full reporting of outcomes
☒	☐	Estimates of effect sizes (e.g. Cohen's *d*, Pearson's *r*), indicating how they were calculated

Our web collection on statistics for biologists contains articles on many of the points above.

Software and code

Policy information about availability of computer code

Data collection	No software was used (data collection was made by Qualtrics).
Data analysis	All analyses were conducted using the R statistical software v. 4.5.0 with JAGS 4.3.2.

For manuscripts utilizing custom algorithms or software that are central to the research but not yet described in published literature, software must be made available to editors and reviewers. We strongly encourage code deposition in a community repository (e.g. GitHub). See the Nature Portfolio guidelines for submitting code & software for further information.

Data

Policy information about availability of data

All manuscripts must include a data availability statement. This statement should provide the following information, where applicable:

- Accession codes, unique identifiers, or web links for publicly available datasets
- A description of any restrictions on data availability
- For clinical datasets or third party data, please ensure that the statement adheres to our policy

Data is available at https://doi.org/10.5061/dryad.w6m905r2k Code and reproducible analysis are available at https://doi.org/10.24433/CO.3129691.v2

Research involving human participants, their data, or biological material

Policy information about studies with human participants or human data. See also policy information about sex, gender (identity/presentation), and sexual orientation and race, ethnicity and racism.

Reporting on sex and gender	Participants answered the following question:<br>- What is your sex? (Male/Female)
Reporting on race, ethnicity, or other socially relevant groupings	Participants answered the following questions:<br>- Enter the year you were born (in YYYY format)<br>- How would you describe your current residence or community? (Large urban area / City / Village / Isolated house)<br>- When it comes to politics, please indicate which of the following you consider yourself. (Extreme left / Left (socio-democrat) / Centre / Right (conservative) / Extreme right)
Population characteristics	See above
Recruitment	Participants were recruited through Qualtrics' national panels
Ethics oversight	The study was approved the Swedish Ethical Review Authority (permit 2022-04276-01)

Note that full information on the approval of the study protocol must also be provided in the manuscript.

Field-specific reporting

Please select the one below that is the best fit for your research. If you are not sure, read the appropriate sections before making your selection.

☐ Life sciences ☒ Behavioural & social sciences ☐ Ecological, evolutionary & environmental sciences

For a reference copy of the document with all sections, see nature.com/documents/nr-reporting-summary-flat.pdf

Behavioural & social sciences study design

All studies must disclose on these points even when the disclosure is negative.

Study description	Quantitative opinion survey of European citizens about large carnivores.
Research sample	We conducted an opinion survey between 2022 and 2023 across all 23 European countries that host breeding populations of large carnivores, using the Qualtrics online survey platform (www.qualtrics.com). A total of 10,807 respondents were recruited through Qualtrics' national panels. All participants provided informed consent prior to participation, and responses were anonymized by Qualtrics before being made available to the research team.
Sampling strategy	For each surveyed country, we aimed to include at least 500 respondents, with a balanced distribution across key demographic categories: an equal number of female and male participants, equal representation of individuals younger and older than the country-specific median age, and an equal number of respondents from rural and urban areas (whether a respondent enters the category rural or urban is determined by Q9, with answers "Village" or "Isolated house" converting to rural, and answers "Large urban area" or "City" converting to urban). This sampling strategy was designed to avoid underrepresentation of demographics that are both harder to reach and potentially influential in shaping large carnivore policy—such as older individuals in rural regions. In a few countries, however, this targeted balance could not be fully achieved. For instance, rural respondents in Bulgaria, Croatia, and Romania were underrepresented relative to our targets.
Data collection	All survey responses were collected online.
Timing	2022-2023
Data exclusions	n/a
Non-participation	The authors were only given the survey responses without details on dropout rate.
Randomization	n/a

Reporting for specific materials, systems and methods

We require information from authors about some types of materials, experimental systems and methods used in many studies. Here, indicate whether each material, system or method listed is relevant to your study. If you are not sure if a list item applies to your research, read the appropriate section before selecting a response.

Materials & experimental systems

n/a	Involved in the study
☒	☐ Antibodies
☒	☐ Eukaryotic cell lines
☒	☐ Palaeontology and archaeology
☒	☐ Animals and other organisms
☒	☐ Clinical data
☒	☐ Dual use research of concern
☒	☐ Plants

Methods

n/a	Involved in the study
☒	☐ ChIP-seq
☒	☐ Flow cytometry
☒	☐ MRI-based neuroimaging

Plants

Seed stocks	*Report on the source of all seed stocks or other plant material used. If applicable, state the seed stock centre and catalogue number. If plant specimens were collected from the field, describe the collection location, date and sampling procedures.*
Novel plant genotypes	*Describe the methods by which all novel plant genotypes were produced. This includes those generated by transgenic approaches, gene editing, chemical/radiation-based mutagenesis and hybridization. For transgenic lines, describe the transformation method, the number of independent lines analyzed and the generation upon which experiments were performed. For gene-edited lines, describe the editor used, the endogenous sequence targeted for editing, the targeting guide RNA sequence (if applicable) and how the editor was applied.*
Authentication	*Describe any authentication procedures for each seed stock used or novel genotype generated. Describe any experiments used to assess the effect of a mutation and, where applicable, how potential secondary effects (e.g. second site T-DNA insertions, mosiacism, off-target gene editing) were examined.*
